# Comparative analysis of ANFIS, ANN, and BBD for enhanced prediction of methyl orange adsorption in water treatment

**DOI:** 10.1038/s41598-026-43445-4

**Published:** 2026-03-09

**Authors:** Simon Bbumba, Ibrahim Karume

**Affiliations:** 1 Department of Chemistry, College of Natural Sciences, Makerere University, P.O. Box 7062, Kampala, Uganda; 2 Department of Science, Faculty of Science and Computing, Ndejje University, P.O. Box 7088, Kampala, Uganda

**Keywords:** Adaptive neural fuzzy inference system, Artificial neural network, Response surface methodology, Activated carbon, Methyl orange, Engineering, Environmental sciences, Mathematics and computing

## Abstract

This work focused on the enhanced prediction of methyl orange removal (MO) from water by activated carbon synthesized from banana peels. Characterization was done using powder X-ray diffraction (PXRD), Fourier transform infrared spectroscopy (FTIR), thermogravimetric analysis (TGA), scanning electron microscopy (SEM), and Brunauer-Emmett-Teller (BET). Modeling and prediction of process variables, pH (5–9), time (3–60 min), and temperature (25–50 °C), was carried out using Box-Behnken design (BBD), artificial neural networks (ANN), and adaptive neuro-fuzzy inference system (ANFIS). Performance metrics of R^2^, Adjusted R^2^, Pearson’s r, mean squared error (MSE), root mean squared error (RMSE), and mean absolute error (MAE) were used to evaluate the models. The regression coefficients from the modeling and prediction showed that BBD (R^2^ = 0.9849), ANFIS (R^2^ = 0.9934), and ANN (R^2^ = 0.9921), which describes the high prediction capacity of the three models. The performance metrics showed that ANFIS had superior capacity in data modeling and prediction compared to BBD and ANN when analyzing complex non-linear relationships. The Elovich, pseudo-first-order, intraparticle diffusion, and pseudo-second-order kinetic models had high R^2^ values. The data obtained showed that the pseudo-second-order fitted the data well; as such, chemisorption was the most dominant mechanism. In addition, the isotherm models of Freundlich, Temkin, Langmuir, and Dubinin-Radushkevich were determined. The Freundlich model shows the highest R^2^, as such adsorption occurs on heterogeneous multilayer surfaces. This study therefore shows the efficiency of ANFIS, ANN, and BBD in the prediction of dye removal by activated carbon synthesized from banana peels.

## Introduction

**Fig. 1:**
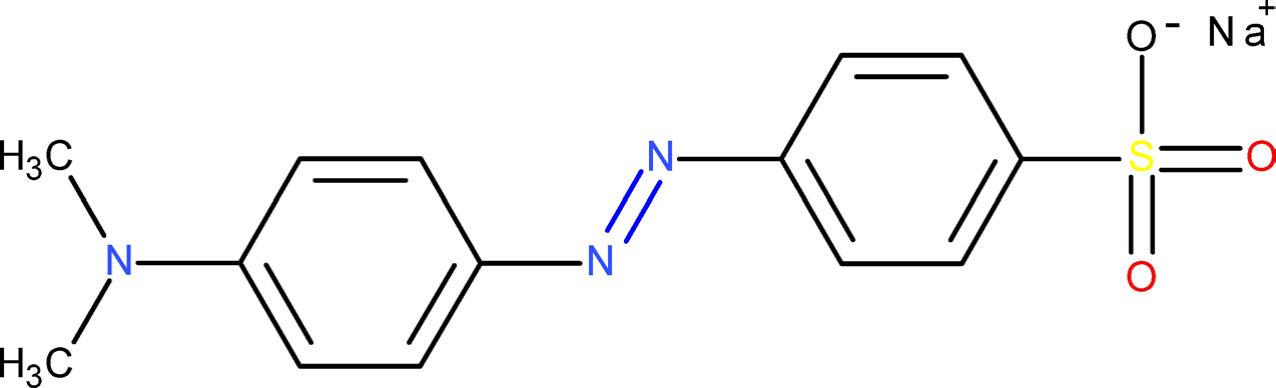
A schematic representation of the methyl orange molecule.

Textile dyes are currently being discharged into the environment due to an increase in population that has resulted in rapid industrialization^1^. The discharge of a large number of dyes into the ecosystem poses a great threat to both humans and aquatic life. These dyes are normally discharged through a variety of sources, such as paper making, clothing, textile industries, analytical labs, and mining^2,3^. They are mainly classified as cationic, anionic, and neutral dyes whose accumulation in the environment causes an increase in toxicity^4^. They cause adverse effects to human beings, such as liver damage, nausea, vomiting, hormonal imbalance, skin, and eye irritations^5,6^. Common examples include methylene blue, rhodamine B, malachite green, crystal violet, methyl orange, alizarin, and methyl red^7^. Among these, methyl orange (MO), which is an azo dye, has been detected in water due to its discharge by the textile industries^8^. MO is highly stable and has a long lifespan; however, it can degrade into more toxic components^9^. Figure 1 shows a representation of the methyl orange molecule.

It disrupts the physicochemical characteristics of the water as it reduces the light penetration into the water, thus disrupting the life under water^10^. MO serves as a representative pollutant among the large class of azo dyes, which are commonly found in industrial wastewater. Due to the presence of the azo group, it acts as a model pollutant that can be used in reference to other dyes. It is thus prudent to develop methods that remove this dye from water^11,12^.

A variety of approaches have been employed for the extraction of methyl orange from water, including membrane filtration^13^, coagulation^14^, electrochemical methods^15^, chemical precipitation, reverse osmosis^16^, advanced oxidation processes^17^, adsorption^13^, and biological treatment^18^. Unfortunately, most of these approaches possess numerous disadvantages, such as fouling in membrane separation, whereas photocatalysis is constrained by reaction duration and the efficacy of the photocatalyst. Moreover, reverse osmosis is a highly costly procedure that consumes significant energy and removes essential minerals from water^19^. The advanced oxidation process incurs substantial operational expenses due to the intricate chemistry of impurities and residual peroxide concentrations^20^. The electrochemical process is significantly influenced by solution pH, whereas coagulation generates a substantial quantity of sludge^21^. Chemical precipitation entails the introduction of chemicals that cannot eliminate colour, resulting in a characteristic potentially hazardous sludge^22^. Nonetheless, adsorption has gained extensive application owing to its straightforward design, affordability, and accessibility of adsorbent materials^23^. It additionally provides the ability to generate minimal sludge and the power to eliminate contaminants at low concentrations.

The efficacy of the adsorption process in eliminating organic and inorganic pollutants from wastewater is contingent upon the choice of adsorbents. Adsorbents are solid materials characterised by active sites on their surfaces, featuring morphological networks of pore distributions, surface charge, and surface functional groups that collectively affect the adsorption process^24^. Nonetheless, the exhausted adsorbents can be reactivated and utilised in subsequent cycles, rendering the adsorption method very sustainable and cost-effective^25^. Various adsorbents have been employed in the literature to illustrate the adsorptive elimination of hazardous dyes from wastewater, including natural clays^26^, biochar^27^, activated carbon from agricultural residues^28^, graphene^29^, carbon nanotubes^30,31^, chitosan^32^, metal-organic frameworks^33^, metal oxides^34^, and their composites.

Activated carbon has been widely acknowledged as an efficient adsorbent for eliminating various pollutants, owing to its remarkable porosity and extensive surface area, hence serving as a fundamental component in water purification methods^35,36^. Nonetheless, the industrial manufacture of activated carbon frequently depends on non-renewable resources such as coal and petroleum, which are not only costly but also exacerbate carbon emissions^37^. This escalating worry has prompted extensive studies into the production of activated carbon from sustainable, economical biomass sources^38^. Activated carbon has been used in the removal of methyl orange due to its large surface area, high porosity, thermostability and cost effectiveness. Banana peels (BP) are wastes that accumulate after the consumption of the food crop, particularly in Africa and most importantly in Uganda^39,40^. They are mainly disposed of as manure in the gardens or as feedstock for animals. It has a high carbon content and as such can be used as an alternative source for activated carbon since it is cheap and readily available. Banana peels are also known to contain a variety of functional groups such as hydroxyl (-OH), amine (-NH_2_), and carboxyl (-COOH) which help to enhance the adsorption capacity of the adsorbent through creation of more binding sites.

Response surface methodology (RSM) is a tool in the prediction and optimization of process variables^41^. It is achieved via the use of input variables which are optimized to produce a response denoted as the output. It is a viable tool for scientists and engineers mainly applied during experimental studies to forecast and optimize an outcome^41,42^. RSM is composed of a variety of models, of which the central composite design (CCD) and the box-behnken design (BBD) are commonly used^43^. They both depend on the number of input variables that influence the output. This study will take into account the BBD to optimize and predict its outcomes. One of its primary advantages is that it takes only a limited number of experimental trials to ascertain the ideal settings. It also offers a statistical approach of confirming the interaction between the input parameters, but also provides the significance of the factors under study. RSM, which is a predefined polynomial, faces some drawbacks when optimizing complex, non-linear adsorption processes; as such, it can be complemented with other advanced artificial intelligence models such as artificial neural networks (ANN) and adaptive neural fuzzy inference system (ANFIS)^44,45^.

Artificial neural networks (ANN) are computer systems that are designed to mimic the function of neurons in living organisms^46^. It has both the input and output layers, which are connected to a number of hidden layers that are mainly achieved via a given study^42^. The network can do internal computations used to derive the desired output from input data. Due to its reliability and efficiency in depicting the nonlinear and complex processes among variables and responses, it can be utilized in various industrial and scientific systems^47,48^. Using the training set, the network can have multiple input and output values for a given non-linear or complex system, which enables it to achieve maximum accuracy^49^. It is very important in giving insights regarding hidden relationships that are sometimes missed by the polynomial form of RSM.

An adaptive neural fuzzy inference system (ANFIS) is a combination of a neural network and a fuzzy system. It is used to simulate and predict non-linear, complex systems and is based on a Sugeno fuzzy inference technique^50^. It gives precise and accurate predictions due to the joining of the fuzzy system and neural network, which gives rise to a hybrid model^51^. The ability for the model to be flexible and dependable is achieved due to having both a fuzzy and a neural network^52^. In recent years, interest in employing ANFIS across many processes has increased^53^. The application of RSM, ANN, and ANFIS was aimed at providing a more comprehensive, comparative, and multifaceted approach that would explain the adsorption process. These models, when used in the adsorption process, complement each other by improving on the single model’s weaknesses, which allows for better optimization and prediction at the different levels. The novelty of this work is based on the application of artificial intelligence models of RSM, ANN, and ANFIS to determine the removal of methyl orange from water by banana peel-activated carbon through understanding the interaction between the input and output process variables. This paper presents modeling and prediction of methyl orange adsorption capacity using BBD-RSM, ANNs, and ANFIS using a readily available raw agricultural material. The results obtained from this study offer an alternative method for the removal of dyes from water, which addresses the Sustainable Development Goals that are vital in better industrial and environmental practices.

## Experimental section

### Materials

Sulphuric acid, sodium hydroxide, distilled water, and nitric acid were all purchased from Sigma Aldrich. Banana peels used in the synthesis of activated carbon were purchased from a market in Kampala district, located in Uganda.

### Adsorbate preparation

**Fig. 2:**
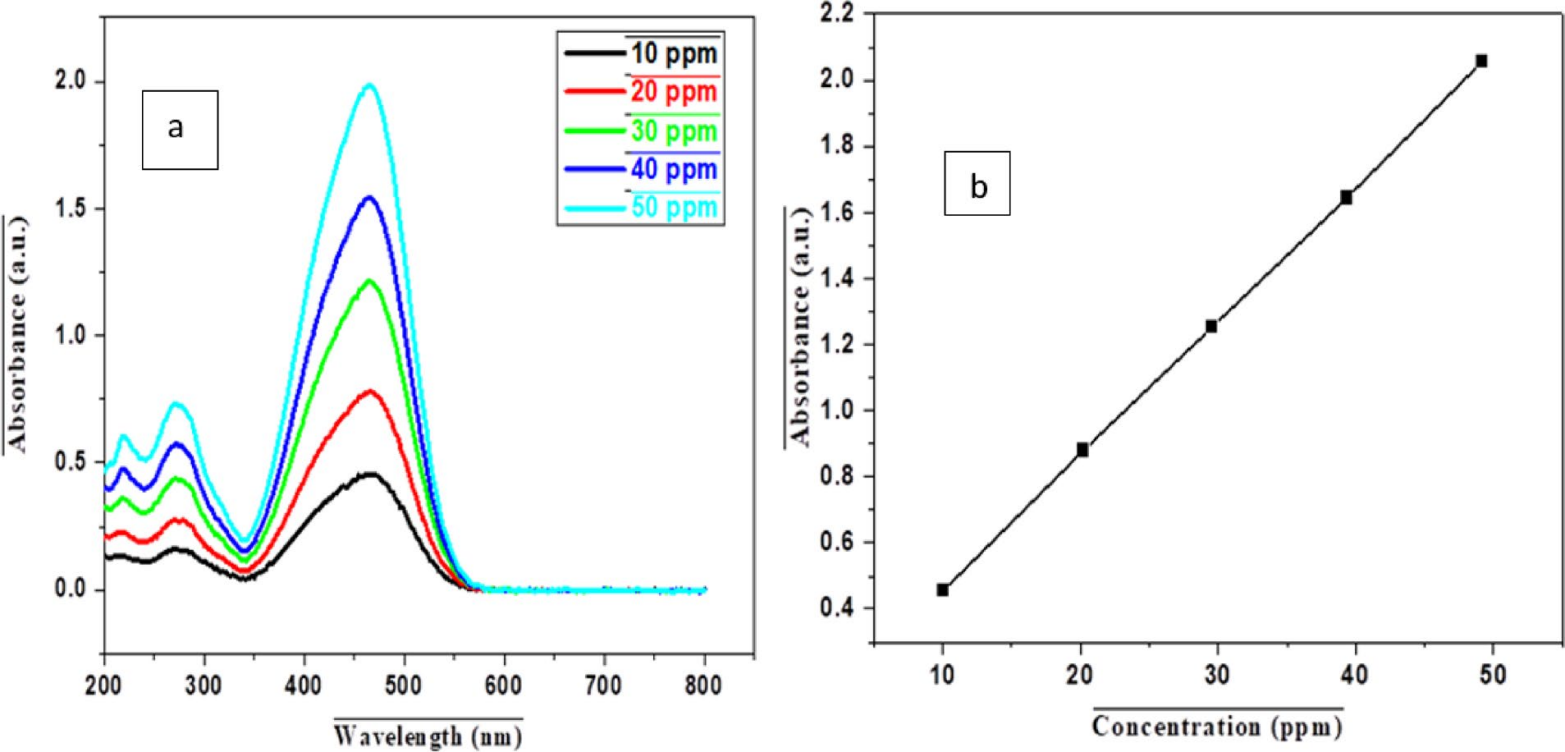
A UV-Vis graph of methyl orange showing absorbance (**a**) and calibration (**b**) plots.

MO, characterized by a dye concentration of 85%, a molecular weight of 327.33 g/mol, and a molecular formula of C_14_H_14_N_3_NaO_3_S, demonstrates maximum absorbance at a wavelength of 460 nm. 1 g of MO was dissolved in 250 mL of distilled water and then diluted to 1000 mL, resulting in 1000 mg/L. Serial dilutions were carried out on the stock solution to achieve solutions with different concentrations, used to draw a calibration curve as shown in Fig. 2.

### Synthesis of banana peel activated carbon

**Fig. 3:**
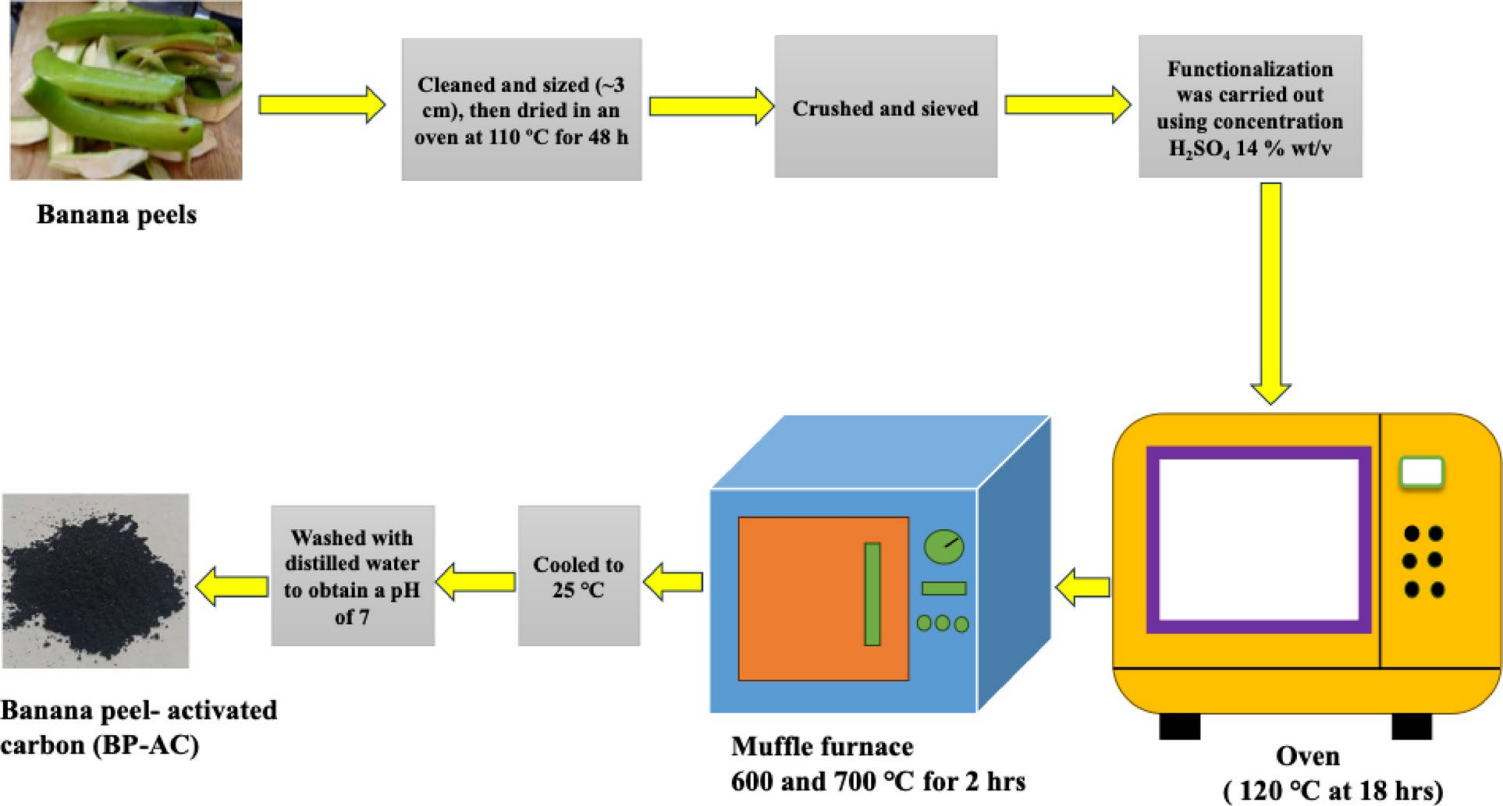
Flow diagram of synthesis of BP-AC.

Activated carbon was synthesized from banana peels (BP) as described by Kigozi et al.^54^ with slight modifications as shown in Fig. 3.

The BP was obtained from Kalerwe market around Kampala. The BP was cleaned and sized (~ 3 cm), then dried in an oven at 110 °C for 48 h. The dried samples were ground and sieved to a fraction using a 1.0 mm sieve. Functionalization of the BP was carried out using a concentration of H_2_SO_4_ 14% wt/v. Activation was achieved at temperatures of 600 and 700 °C, respectively. The obtained activated carbon was washed with distilled water until a pH of 7 was obtained.

### Characterization of the adsorbent

Characterization was carried out using various analytical techniques, including powder X-ray diffraction (PXRD), Fourier transform infrared spectroscopy (FTIR), thermogravimetric analysis (TGA), scanning electron microscopy (SEM), and Brunauer-Emmett-Teller (BET).

### Thermogravimetric analysis

This analysis was conducted on the raw material utilizing the TGA-DSC analyzer (Jupiter STA449 F3 NTZSCH GmbH) from Selb, Germany. Nitrogen was selected as the oxidizing atmosphere for conducting the thermogravimetric analysis. The temperature program and the associated mass versus temperature curve are documented within a computer data system. 1 gram of the material was placed in a sample holder and inserted into an autosampler. The temperature program was configured to range from 28 °C to 1000 °C, utilizing a flow rate of 300 mL/min and a ramped temperature increase of 3 °C/min.

### Scanning electron microscopy

The morphological analysis of the banana peel-activated carbon was conducted using a scanning electron microscope (Gemini-SEM 500 M/s Carl ZEISS-EDAX Z2 Analyzer AMETEK). Before microscopy, the specimens underwent a sputter-coating process using gold with the Edwards Model S150B Sputter Coater. Following this, the specimens were captured through photography, and an analysis of the microstructure of BP-AC was conducted.

### Fourier transformation infrared spectroscopy (FT-IR)

The chemical structure of activated carbon and silica xerogel was analyzed using Fourier transform infrared spectroscopy. In this measurement, the pulverized samples were thoroughly combined with KBr powder, and the background noise was adjusted using pure KBr. FT IR spectra were obtained within the range of 4000 to 500 cm^− 1^, utilizing 32 scans per spectrum and a resolution of 2 cm^− 1^. Transmittance data were plotted against wave number in cm^− 1^.

### Powder X-ray diffraction

The crystallinity of activated carbon was examined using an X-ray diffractometer (RIGAKU Corp., Tokyo, Japan) that utilized Cu kα radiation (wavelength λ = 1.54 Å) alongside the JCPDS-ICDD database. The samples were meticulously crushed into a fine powder, and a uniform layer was carefully positioned in the holder hole using transparent cello tape before being placed in the designated sample position. The measurements were conducted in transmission mode, spanning from 20 ° to 105 ° 2 θ, utilizing a step width of 0.9 ° and an irradiation duration of 10 s.

### Brunauer-Emmet-Teller (BET)

The specific surface area of activated carbon was assessed utilizing BET (11-2370. Gemini Micromeritics, Atlanta, GA, USA). The determination is typically performed at liquid nitrogen temperature. The samples underwent crushing and degassing at a temperature of 200 °C. The calculation of the specific surface area was conducted using Eq. 1^55^.

1 $$\frac{1}{\mathrm{v}\left[\right(\mathrm{P}\mathrm{o}/\mathrm{P})-1]}=\frac{\mathrm{C}-1}{{\mathrm{v}}_{\mathrm{m}}\mathrm{C}}\left(\frac{\mathrm{P}}{{\mathrm{P}}_{\mathrm{o}}}\right)+\frac{1}{{\mathrm{v}}_{\mathrm{m}}\mathrm{c}}$$

The BET constant is represented by c, while P denotes the equilibrium pressure. Po indicates the saturation pressure of the adsorbates at the adsorption temperature. V refers to the quantity of gas that has been adsorbed, and Vm signifies the quantity of gas adsorbed in a monolayer.

### BBD- optimization

The optimization procedure for the removal of MO dye using activated carbon from the solution was designed using BBD with three independent variables, leading to 17 experimental runs. The variables under investigation included time (A), temperature (B), and pH of the MO dye solution (C), with their corresponding ranges detailed in Table 1. The time was varied from 3 to 60 min to determine the rapid adsorption of the adsorbate by the adsorbent within that given range. Temperature was determined starting from 25 to 50 °C to understand the effects at both room and moderately ambient conditions to provide insights on whether the process is exothermic or endothermic. Lastly, pH was carried out in the range of 5–9 to determine the influence of surface charge on the adsorption of the adsorbate, but also to understand the optimum for the electrostatic interactions. The collected data were related to the adsorption capacity. The ranges of the independent variable were determined by considering the point of zero charge of the adsorbent and employing the dose optimization method. The optimization process was carried out using the Design Expert (Stat-Ease, DX11) software, version 13.0.

**Table 1:** Box behnken design process variables.

Entry	Code	Factors	Methyl orange		
			-1	0	+ 1
1	A	Temperature (°C)	25	37.5	50
2	B	Time (minutes)	3	31.5	60
3	C	pH	5	7	9

### Artificial neural networks

**Fig. 4:**
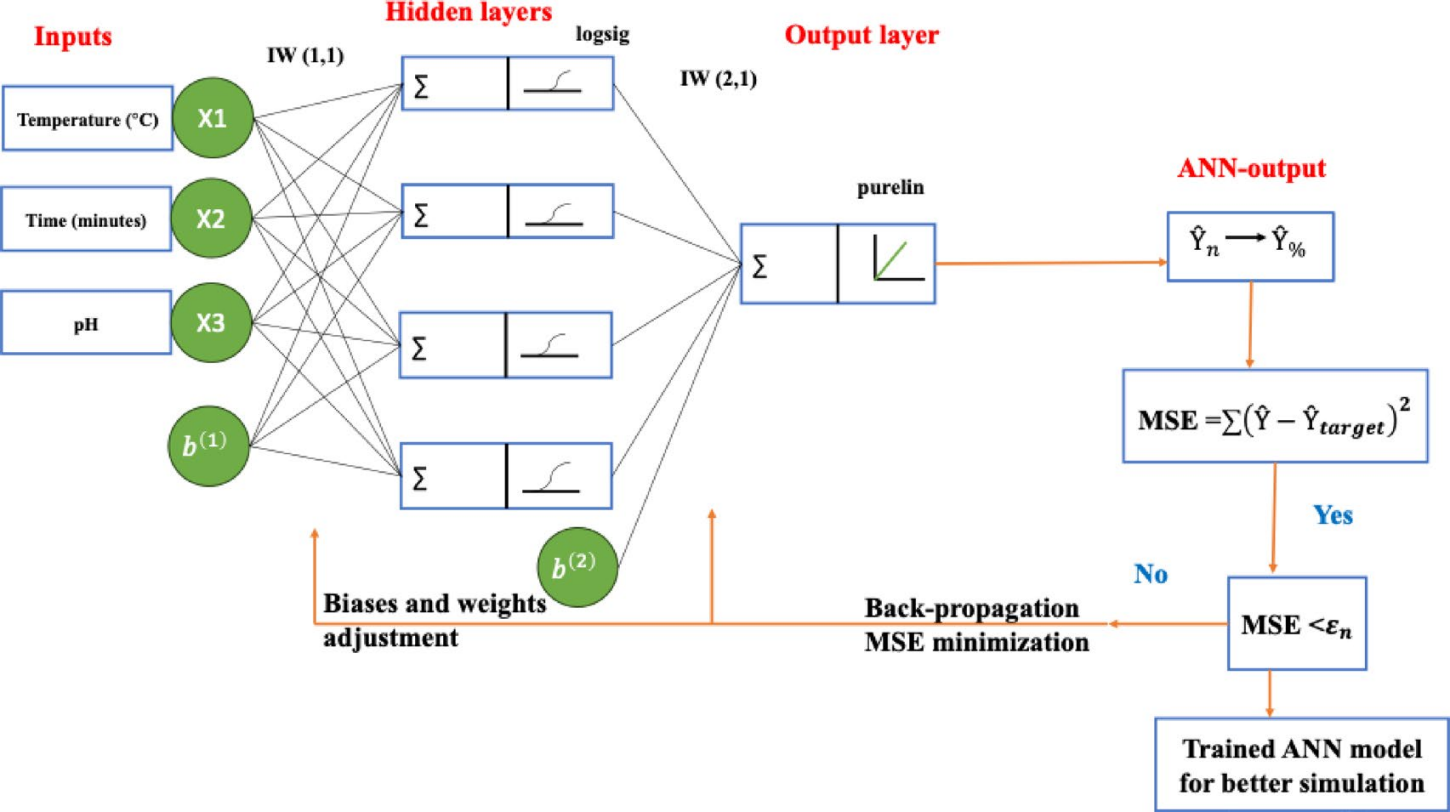
A basic architecture of an artificial neural network.

Neurons are considered essential components of the network, and they are interconnected. Every network consists of neurons structured in layers, linked through weights. An architecture was developed for this study, as shown in Fig. 4.

The neurons are basic components of the neural network, and they transport information using the weights, which move information in the form of signals. Hidden layers are used in the preprocessing of the data, which is moved to the additional layers and the output through the application of the transfer functions. The input data that was used in the neural network was adopted from the BBD design, which was aimed at having a more elaborate comparison of the input and output variables. In this study, the ANN model was applied using MATLAB R2016b, version 9.1.0, https://www.mathworks.com.

### Adaptive neural fuzzy inference system

**Fig. 5:**
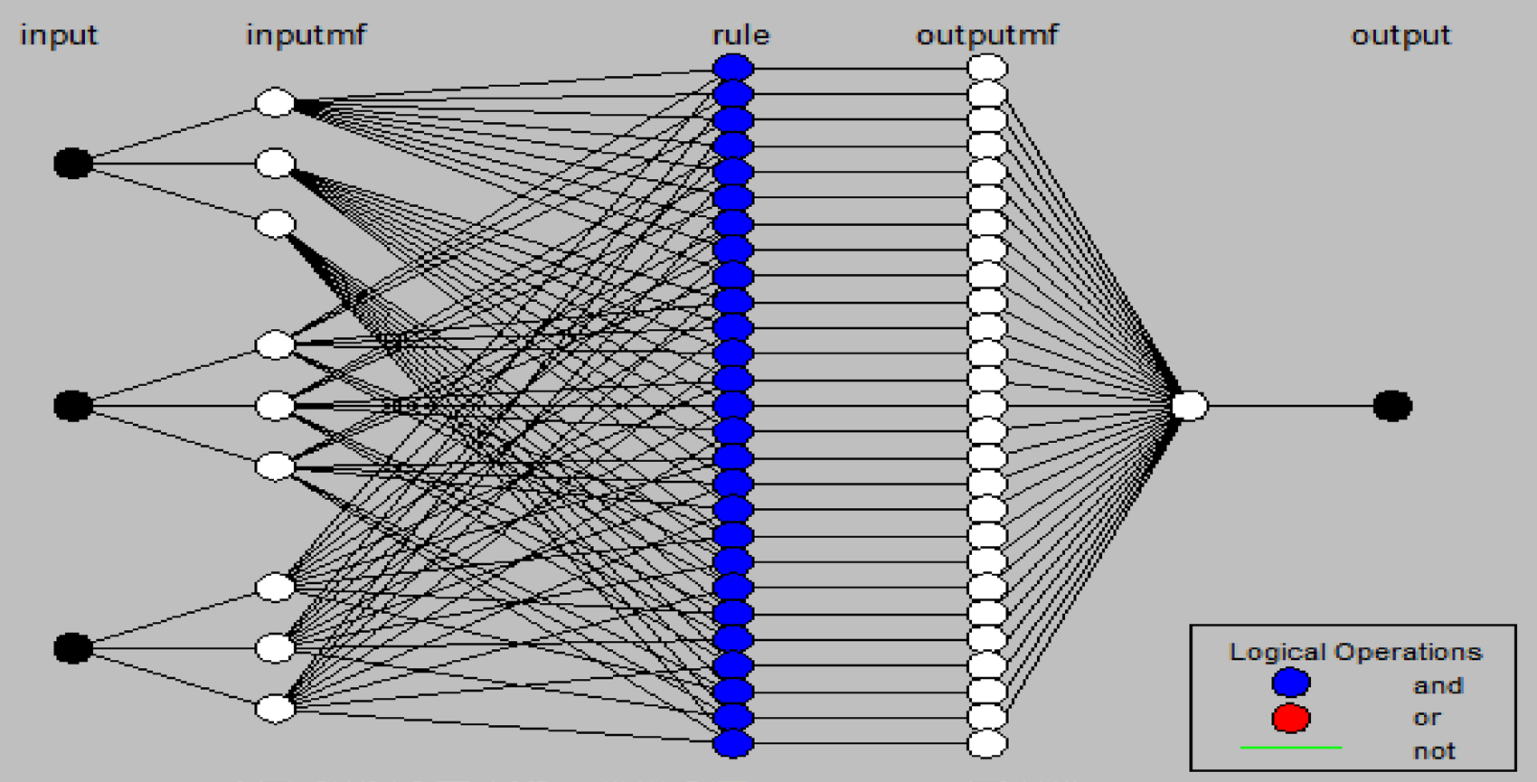
A basic ANFIS structure showing the different layers.

The values utilized in the ANN were uniformly implemented in the modeling. It was executed as a five-layer neural network, employing the foundational concepts of fuzzy inference systems^56^. Figure 5 illustrates the ANFIS structure, where the initial and terminal layers correspond to (temperature, time, dose, and pH) as the inputs and (adsorption capacity) as the output.

It corresponds with Sugeno inference systems based on a first-order, which uses a membership function (MF) after the conversion of the input variables through the method of fuzzification^57^. The ANFIS model for the removal of methyl orange was constructed using MATLAB R2016b, version 9.1.0, https://www.mathworks.com.

### Kinetic and isotherm models

The adsorption mechanism of the synthesised BP-AC for methyl orange was investigated using five kinetic models: Elovich, pseudo-first-order (PFO), pseudo-second-order (PSO), Fractional Power, and intraparticle diffusion (IPD). The models and their corresponding non-linear equations are presented in Table 2.

**Table 2:** The kinetic models studied during tetracycline removal.

Entry	Kinetic model	Equation	Reference
1	PFO	${q}_{t}={q}_{e}\left(1-{e}^{-{k}_{1}t}\right)$	^58^
2	Elovich	${q}_{t}=\frac{{q}_{e}^{2}{k}_{2}t}{1+{q}_{e}{k}_{2}t}$	^59^
3	PSO	${q}_{t}=\frac{{q}_{e}^{2}{k}_{2}t}{\left(1+{q}_{e}{k}_{2}t\right)}$	^60^
4	IPD	${q}_{t}={K}_{id}{t}^{0.5}+{C}_{i}$	^61^
5	Fractional power	${q}_{t}=K{t}^{v}$	^62^

q_e_ (adsorption at equilibrium uptake), q_t_ (adsorption uptake at a given time). K_1_ (first-order rate constant) t (time). C_i_ (thickness of the boundary layer), K_id_ (constant of diffusion), K_2_ (coefficient of second-order rate), β (constant of desorption), and α (adsorption rate).

Furthermore, the non-linear isotherm models based on the classification were used to characterize MO adsorption at equilibrium. The parameters for these models, which describe the adsorption process, are presented in Table 3.

**Table 3:** Adsorption isotherm models studied during methyl orange removal.

Entry	Isotherm model	Equation	Ref
1	Langmuir	${q}_{e}=\frac{{q}_{m}{K}_{L}{C}_{e}}{1+{K}_{L}{C}_{e}}$	^63^
2	Temkin	${q}_{e}=\frac{RT}{{\Delta}\mathrm{q}}In\left(A{C}_{e}\right)$	^64^
3	Freundlich	$${q}_{e}={K}_{f}{C}_{e}^{\raisebox{1ex}{$1$}\!\left/\!\raisebox{-1ex}{$n$}\right.}$$	^65^
4	Dubinin Radushkevich	${q}_{e}={q}_{m}exp\left(-\beta{\epsilon}^{2}\right)$	^66^

q_e_ (adsorption at equilibrium uptake), k_f_ (Freundlich constant related to adsorption uptake), and n (adsorption intensity). A_T_ (intercept derived constant), B_T_ (Temkin isotherm), β (Dubinin-Radushkevich constant), ε (Polanyi constant), and q_m_ (adsorption uptake at maximum).

**Fig. 6:**
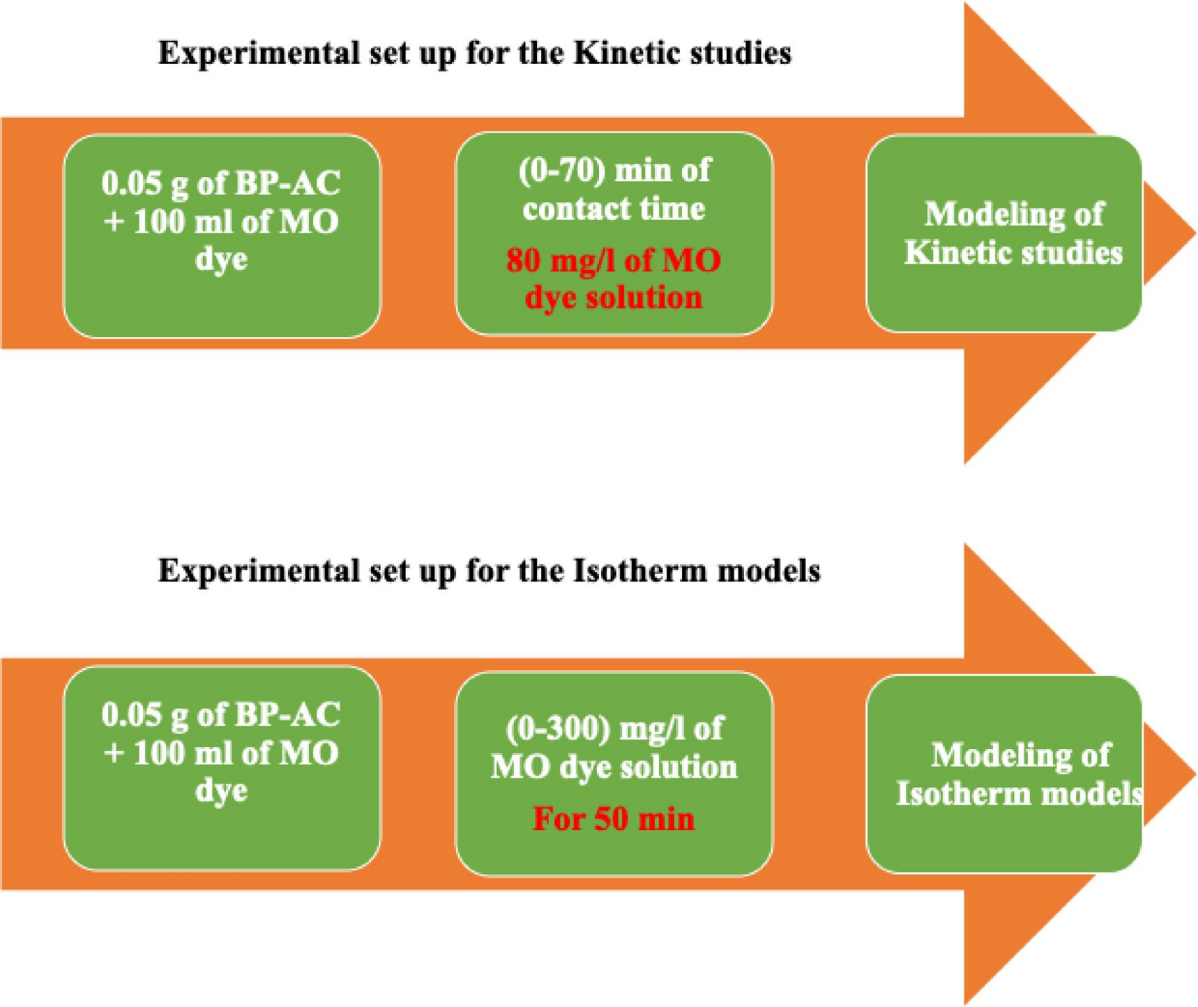
Experimental set up for kinetic and isotherm models.

Finally, the experimental configuration for the adsorption process pertaining to both the kinetic and the isotherm models is illustrated in Fig. 6.

The analysis for the construction of adsorption kinetics and isotherms for removal of methyl orange was performed using Origin Pro, Version 8.5.0, www.originlab.com.

## Results

**Fig. 7:**
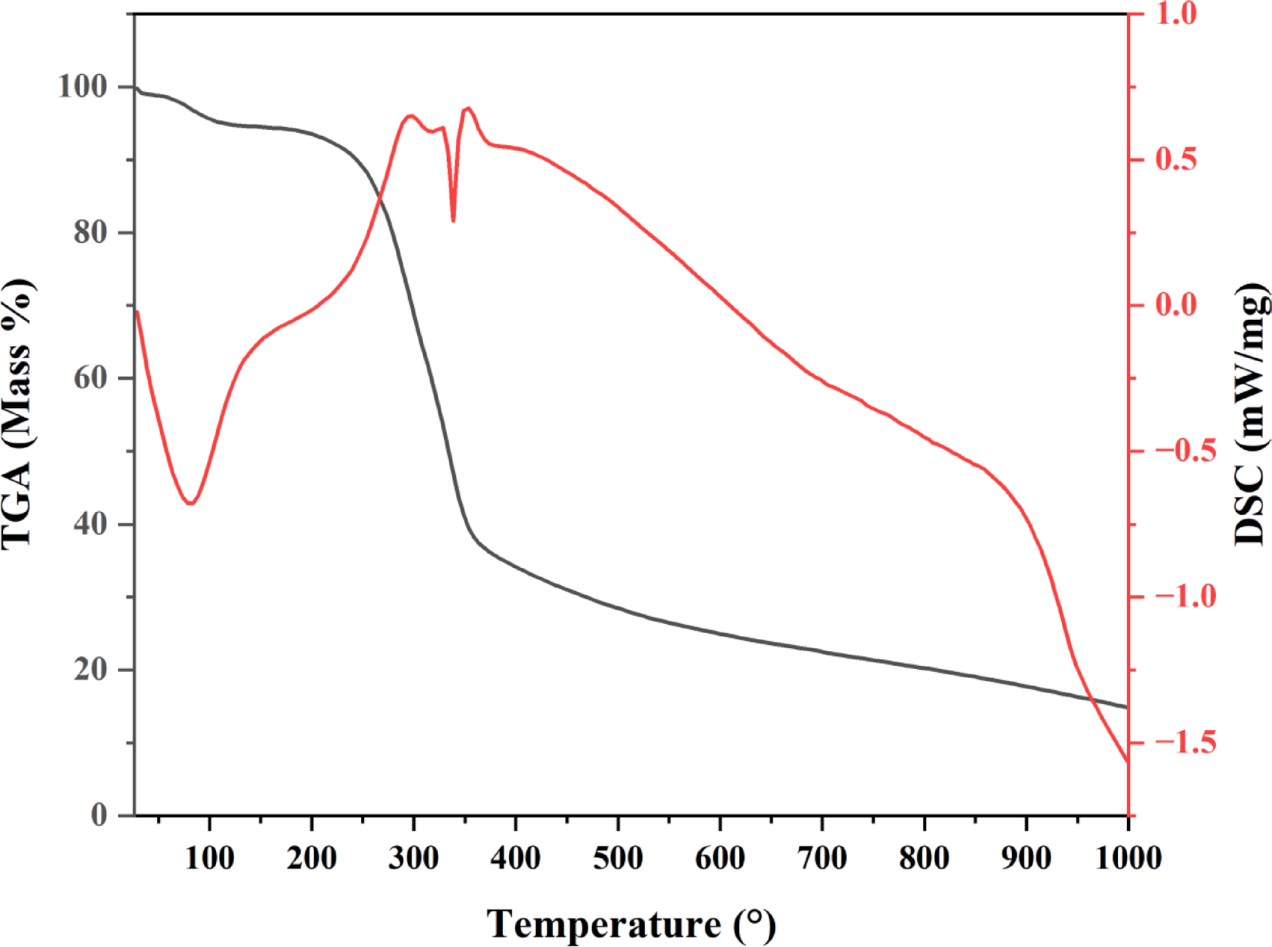
The percentages of different components and analysis profile of banana peel powder.

Figure 7 illustrates the percentage composition of the banana peel powder. The analysis of banana peels reveals two primary phases of weight reduction. The initial phase occurs at temperatures between 27 and 98 °C on the TGA graph, attributed to the moisture loss from the powder. The second stage was noted between 200 and 500 °C at 72.45%, attributed to the breakdown of volatile organic matter and surface functional groups like carboxyl^67^. The maximum mass loss observed at 500 °C indicates that this temperature is optimal for carbonization. The activation of the material commenced at 600 °C, at which point the material exhibited a stable mass. The elevated decomposition temperature of banana peels demonstrates their durability for applications involving high temperatures.

**Fig. 8:**
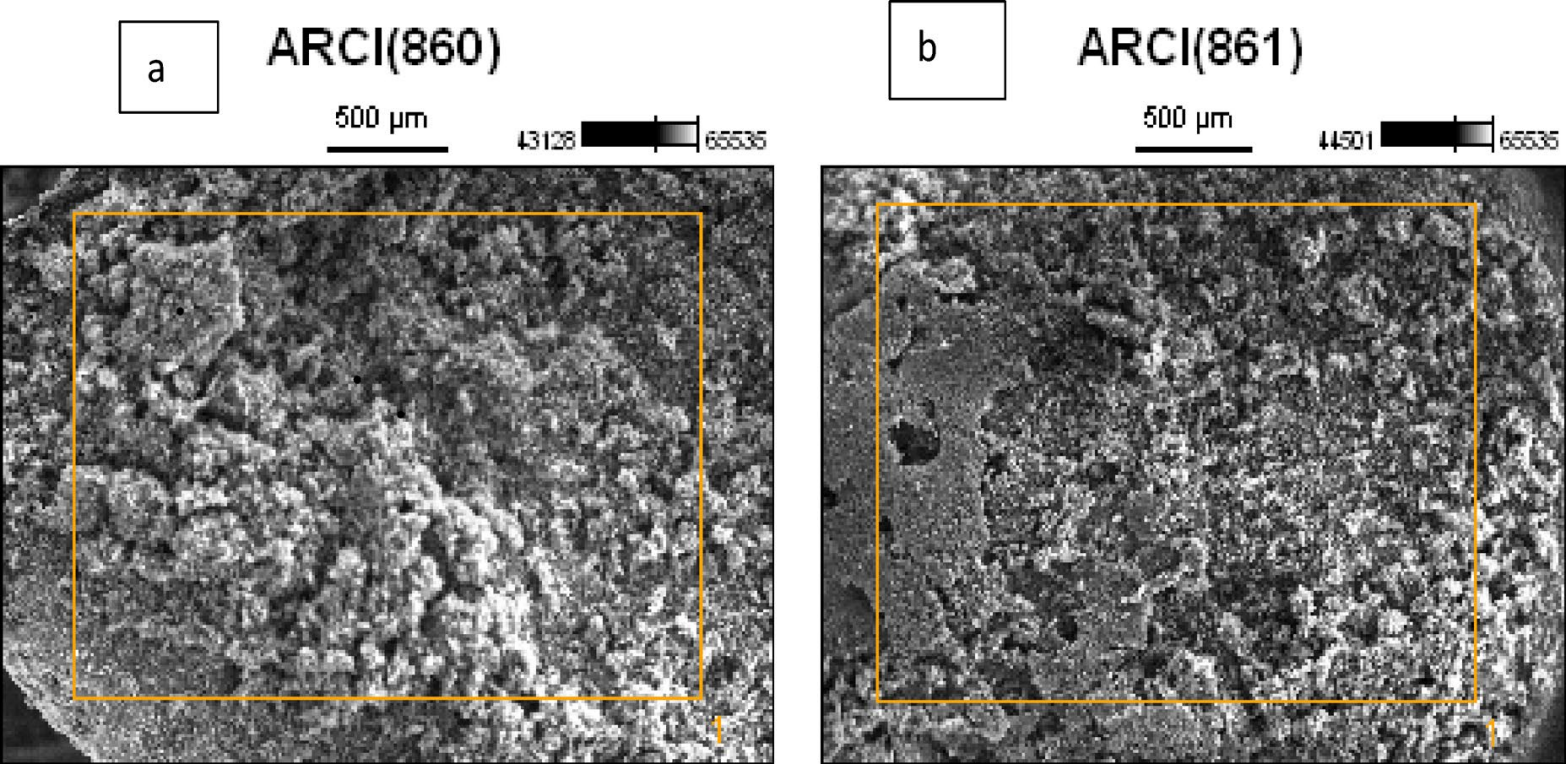
Scanning electron microscopy images of BP powder at 600 and 700 °C.

Figure 8 presents SEM images of banana peel-activated carbon at two activation temperatures (600 and 700 °C). The activated carbon samples a and b produced at activation temperatures of 600 and 700 °C exhibit varying pore sizes that are associated with the removal of volatile organic compounds during the carbonization process. The particles exhibited a rough surface, likely resulting from extensive activation processes.

**Fig. 9:**
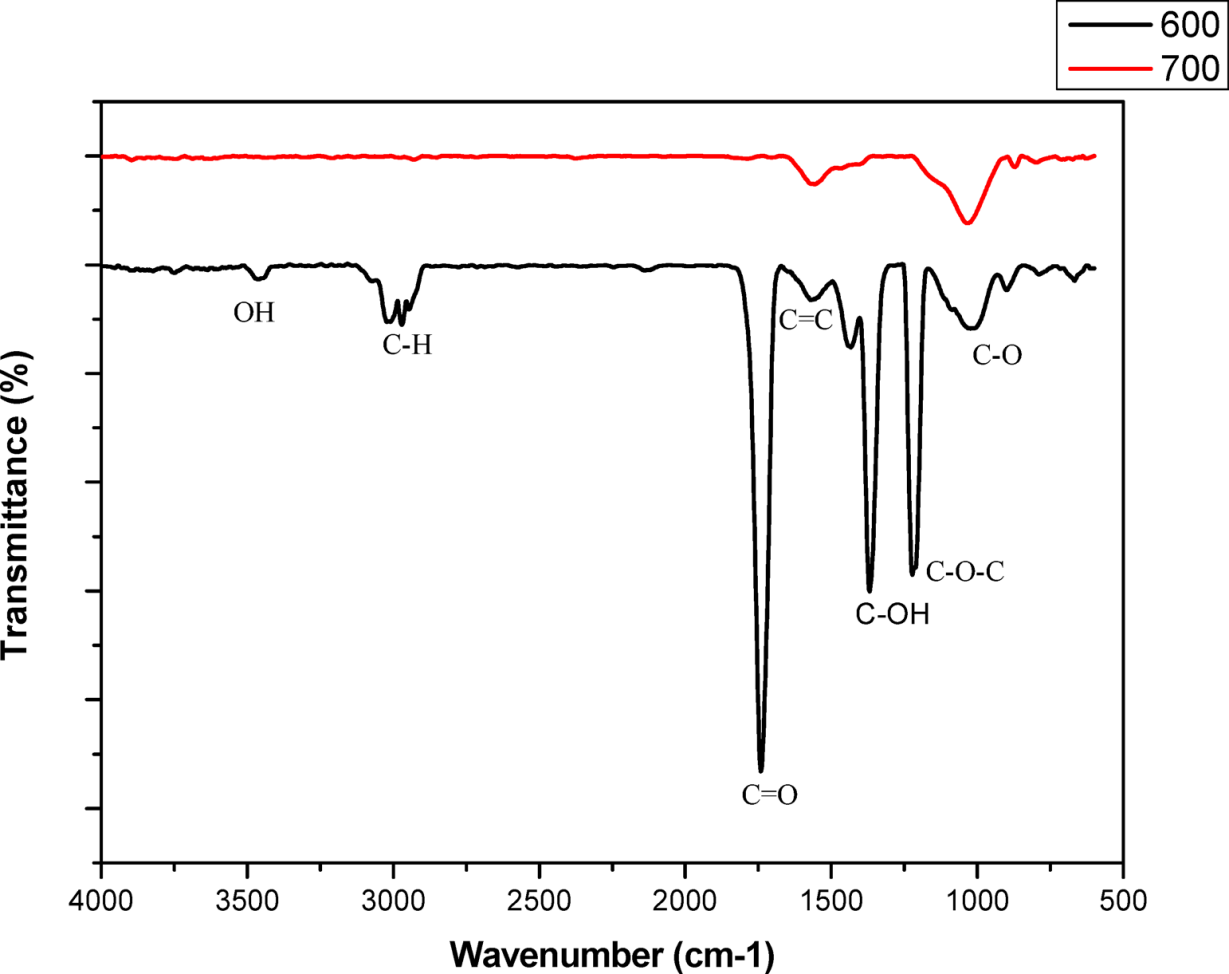
The FTIR spectrum of the activated carbon.

Figure 9 shows the FTIR spectra for activated carbon at 600 and 700 °C, respectively. The two samples showed peaks within the same regions of the spectrum. The peaks corresponding to C-O, C-C, C-N, and ^−^OH stretches were observed in the 1000–1500 cm^− 1^ region, which are characteristic of the fingerprint. The peaks at 1070, 1220, 1700, and 3000 cm^− 1^ are attributed to C-C and C-O, C = O, and C–H sp^3^ stretching, respectively. Materials such as cotton stalks^68^, olive stones^69^, and rice husks^70^ have also shown similar peaks.

**Fig. 10:**
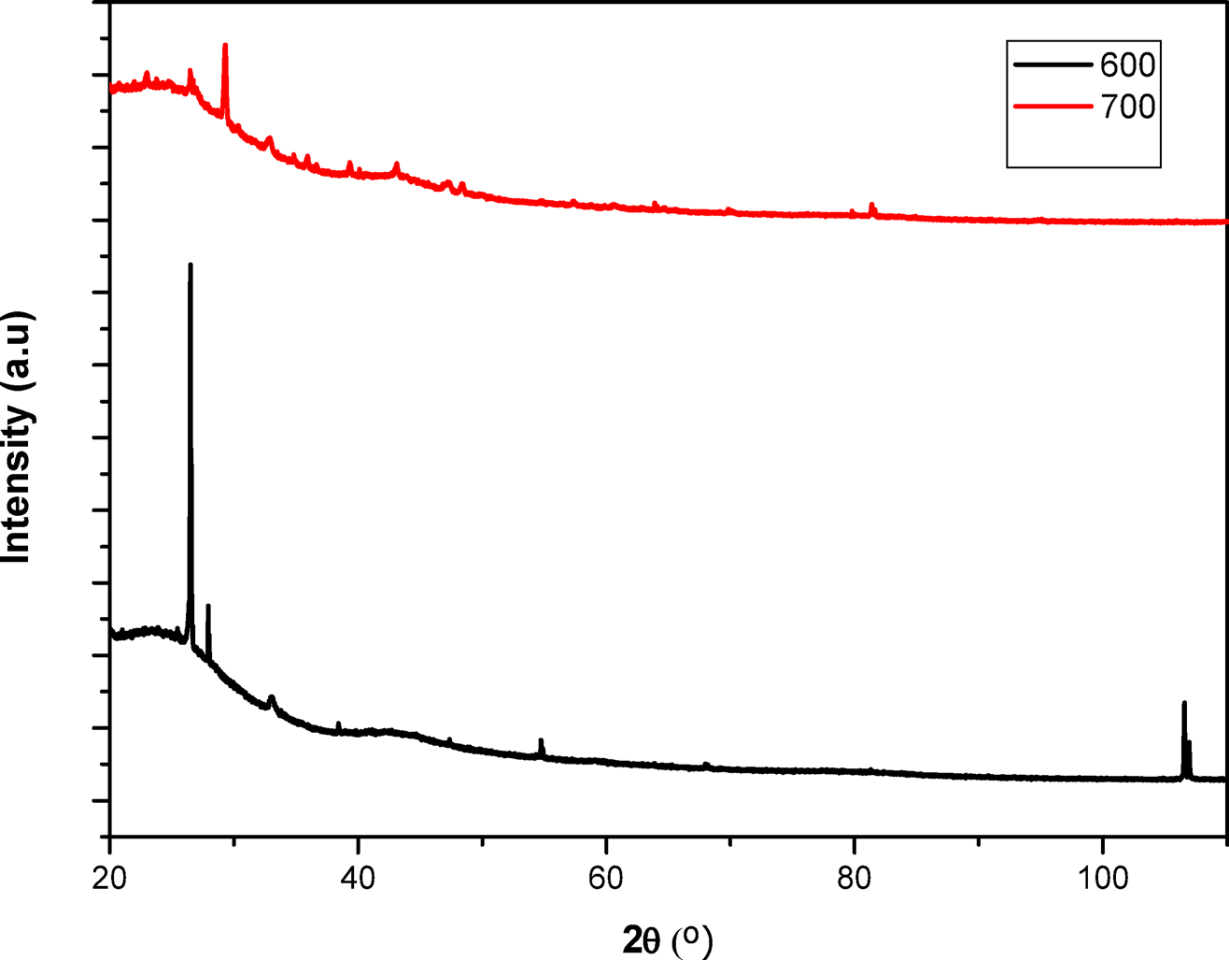
The XRD spectrum of the activated carbon.

Figure 10 shows the powder XRD spectra of the activated carbon. The two samples showed peaks in the 2θ range of 23 ° to 30 ° and 40 °to 47 °, which evidence materials with graphite-like structures. The crystal planes of (002) and (110), along with the presence of bands around 2θ of 24° and 44°, show BP-AC materials with amorphous and non-crystalline structure^71^.

**Fig. 11:**
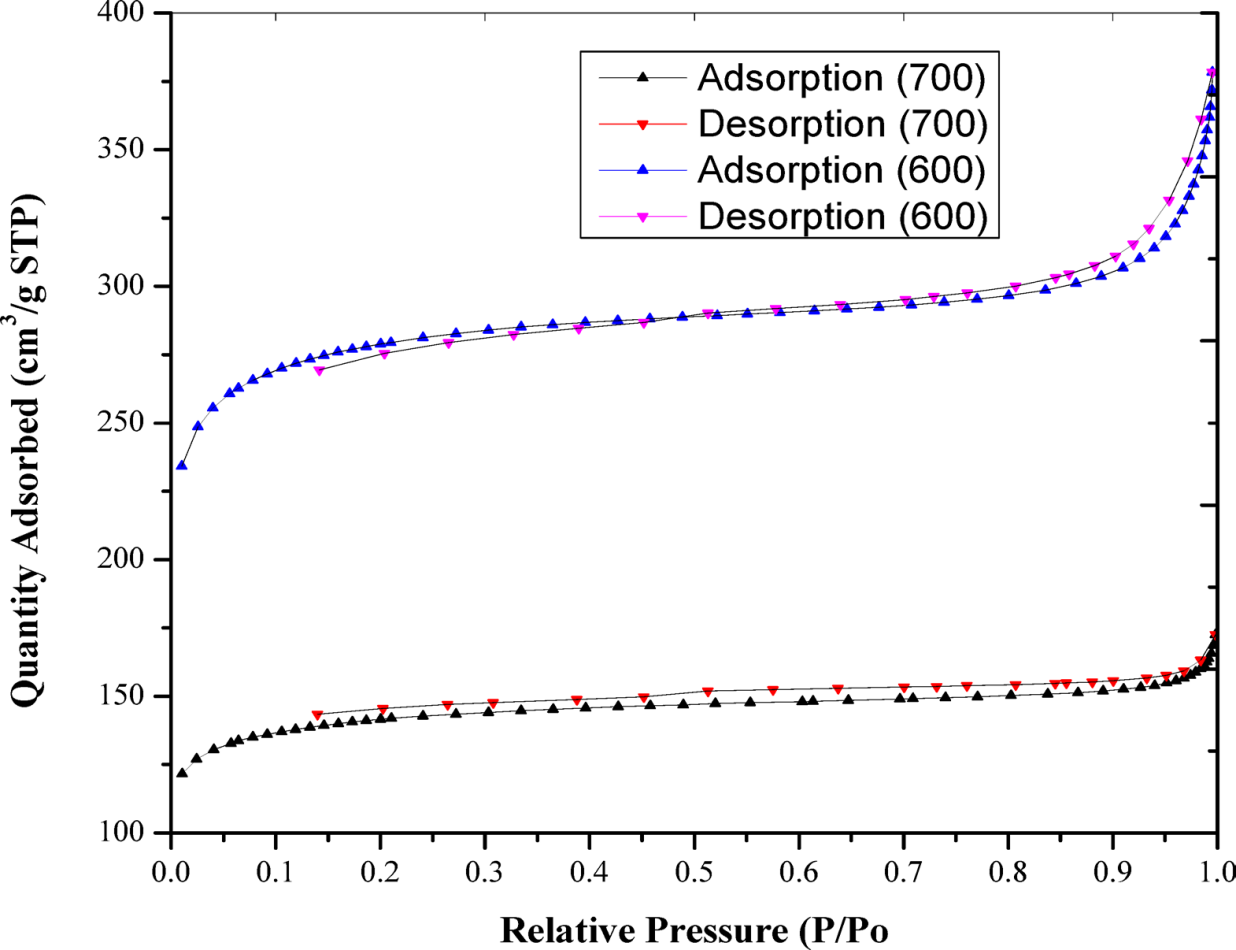
The N_2_ adsorption/desorption of the activated carbon.

The physical properties of the activated carbon were assessed through N_2_ adsorption-desorption, as illustrated in Fig. 11. The technique revealed a BET surface area S_BET_ of 253.50 m^2^/g for AC-600 and 104.22 m^2^/g for AC-700.

The specific surface area and pore volume diminished as the activation temperature for the two samples increased. The surface area diminishes with rising temperature, and a comparable pattern is noted for the micropore volume, which also decreases as the temperature increases. This suggests that materials treated with H_2_SO_4_ acid exhibited low physical properties while demonstrating enhanced chemical properties, such as functionalization, as the activation temperature increased. The acid-induced oxidation and hydrolysis reactions, with the effect increasing at high temperatures. This phenomenon results in the obstruction of both the mesopores and micropores of the activated carbon. The isothermal curves featuring hysteresis loops represent type I and IV curves.

### BBD-model selection

The models commonly selected include quadratic, cubic, two factors (2F), and linear forms, which depend on the best performance prediction for the adsorption capacity of MO, but also significant power towards the data. The models were examined, with the findings presented in Table 4. The quadratic model was selected as the best equation to study the adsorption of MO from water, as it gave a high R^2^ and a p-value lower than 0.05 compared to the other equations.

**Table 4:** Analysis of the model equations.

Source	Sequential *p*-value	Lack of Fit *p*-value	Adjusted *R*²	Predicted *R*²	
Linear	0.0005	0.0070	0.6692	0.5814	
2FI	0.7743	0.0043	0.6132	0.3567	
**Quadratic**	**0.0001**	**0.2969**	**0.9655**	**0.8529**	**Suggested**
Cubic	0.2969		0.9738		**Aliased**

2 $$\begin{aligned} {\text{Methyl Orange removal }} & = 146.378{\text{ }} + {\text{ }}30.2987{\text{ }}*{\text{ }}A{\text{ }} + {\text{ }}11.3963{\text{ }}*{\text{ }}B{\text{ }} + {\text{ }} - 48.5475{\text{ }}*{\text{ }}C{\text{ }} + {\text{ }} - 13.975{\text{ }}* \\ & AB{\text{ }} + {\text{ }}6.4275{\text{ }}*{\text{ }}AC{\text{ }} + {\text{ }}3.9225{\text{ }}*{\text{ }}BC{\text{ }} + {\text{ }} - 20.2065{\text{ }}*{\text{ }}A^{2} + {\text{ }} - \\ & 14.1965{\text{ }}*{\text{ }}B^{2} + {\text{ }} - 34.899{\text{ }}*{\text{ }}C^{2} \\ \end{aligned}$$

Equation 2 incorporates the input variables based on the actual values, which include pH, time, and temperature. It also considers their interactions (pH-time, pH-temperature, and time-temperature as well as quadratic terms (pH², time², and temperature²) to illustrate the adsorption of methyl orange, thereby elucidating the influence of each parameter at practical levels. The coefficients, both positive and negative, illustrated in the preceding equation, indicate the combined effect of the different parameters on the adsorption capacity. The coefficients exhibiting positive and negative values influence the response positively and adversely, respectively.

### ANOVA for the quadratic model

It was used to investigate the best model factors. This analysis was used to clearly show the interactive and individual effects of the input variables on the adsorption capacity. ANOVA analysis is generally used as a means of validating the suggested model. In addition, the quadratic model was employed to show variations in the model derived from statistics, but also to describe the factors with the greatest importance.

This study examined the impact of three distinct parameters on the adsorption capacity using ANOVA, with the results presented in Table 5.

**Table 5:** Statistical analysis of the input variables.

Source	Sum of Squares	df	Mean Square	F-value	*p*-value	
**Model**	36687.18	9	4076.35	50.69	< 0.0001	significant
A-Time	7344.11	1	7344.11	91.33	< 0.0001	
B-Temperature	1039.00	1	1039.00	12.92	0.0088	
C-pH	18854.88	1	18854.88	234.47	< 0.0001	
AB	781.20	1	781.20	9.71	0.0169	
AC	165.25	1	165.25	2.05	0.1948	
BC	61.54	1	61.54	0.7653	0.4107	
A²	1719.17	1	1719.17	21.38	0.0024	
B²	848.59	1	848.59	10.55	0.0141	
C²	5128.17	1	5128.17	63.77	< 0.0001	
**Residual**	562.91	7	80.42			
Lack of Fit	318.65	3	106.22	1.74	0.2969	not significant
Pure Error	244.26	4	61.06			
**Cor Total**	37250.09	16				

The quadratic model for predicting adsorption capacity as the response was assessed via statistical analysis, as shown in Table 5, which included the P-value, lack of fit, and F-value. ANOVA delineates components including SS, DF, and MS. The F-value of 14.55 indicates that the model exhibits statistical significance. The chances of the F-value being obtained exclusively from random variation are about 0.01%. Values of “Probability > F” below 0.0500 signify the relevance of the study. The data demonstrated that significant model variables are A, B, and D; however, the terms AB, BC, AC, A², C², and B² lack importance. Moreover, the terms with values over 0.1000 demonstrated insignificance.

**Fig. 12:**
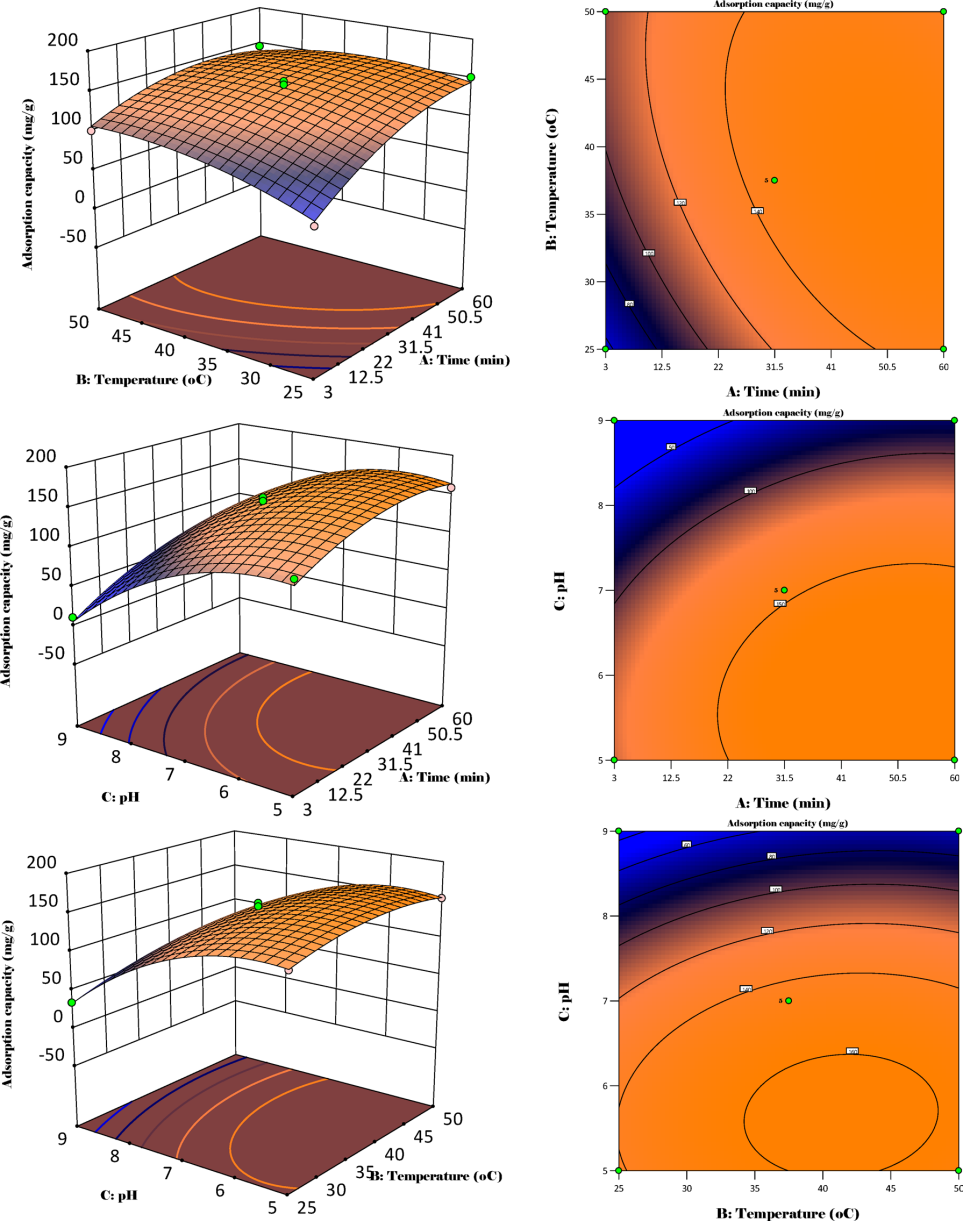
3D and 2D surface and contour plots showing process variable interaction.

3D and 2D graphs illustrating the interaction of the three parameters on adsorption capacity were also discussed as shown in Fig. 12.

Figure 12 illustrates 3D and 2D contour and surface plots for the three input variables as functions of the output. Initially, the adsorption capacity greatly increases as temperature and time increase, which signifies that the process could be endothermic. This is mainly due to the time of interaction between the adsorbate and the adsorbent, due to interaction in the micropores, but also the increase in kinetic energy of the molecules, which leads to higher interactions. It is also observed that a decrease in the activation energy leads to a high reaction rate, which is further responsible for the observed high adsorption capacity^72^. Furthermore, the effect of pH and time on the adsorption capacity is investigated. The adsorption capacity increases at lower pH levels but with higher contact times, which is attributed to the acidic nature of the adsorbent surface that allows strong attraction for the adsorbate. It is also evident that the number of surface functional groups increases, which leads to a number of interactions such as hydrogen bonding and electrostatic interaction. The duration of the interaction of the adsorbent and adsorbate at these pH conditions is also a significant factor that greatly affects the adsorption capacity. Lastly, the interaction between pH and temperature is also investigated. At low pH and slightly higher temperatures, the adsorption capacity increases due to the increase in the surface charge and interaction of the adsorbate on the micropores of the adsorbent. At high temperatures and pH, the adsorption capacity decreases due to the strong repulsion on the surface of the adsorbent, but also the lack of enough surface functional groups, whose degradation is assumed to occur at elevated conditions. This study, therefore, aligns with previous studies that made similar observations regarding the improvement of adsorption capacity at lower pH (slightly acidic), high contact time, and medium temperature conditions.

### The normal probability plot of the residuals

**Fig. 13:**
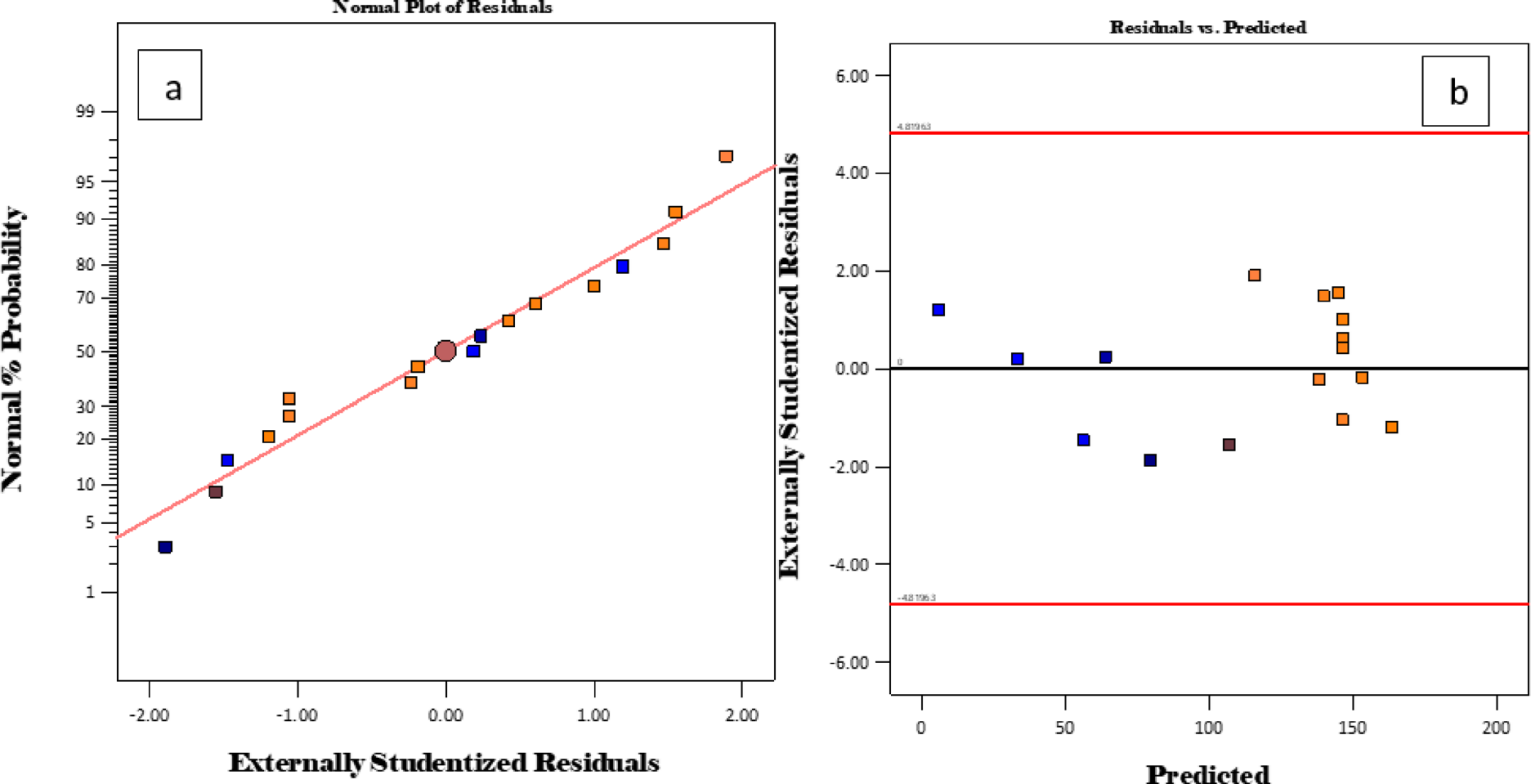
Plots of actual, predicted, and normal charts of the residuals.

Figure 13 displays the plots for actual, expected, and normal charts of the residual associated with the experimental data in the investigation of adsorption capacity.

Figure 13a shows a linear correlation in the data obtained, signifying that output relates to the predicted values. In addition, Fig. 13b shows that the fluctuation of the studentized residuals in comparison to the projected values lacks any discernible pattern. Consequently, it may be concluded that the acquired residuals demonstrate standard variability and substantiate the legitimacy of the suggested model.

### Model adequacy

**Fig. 14:**
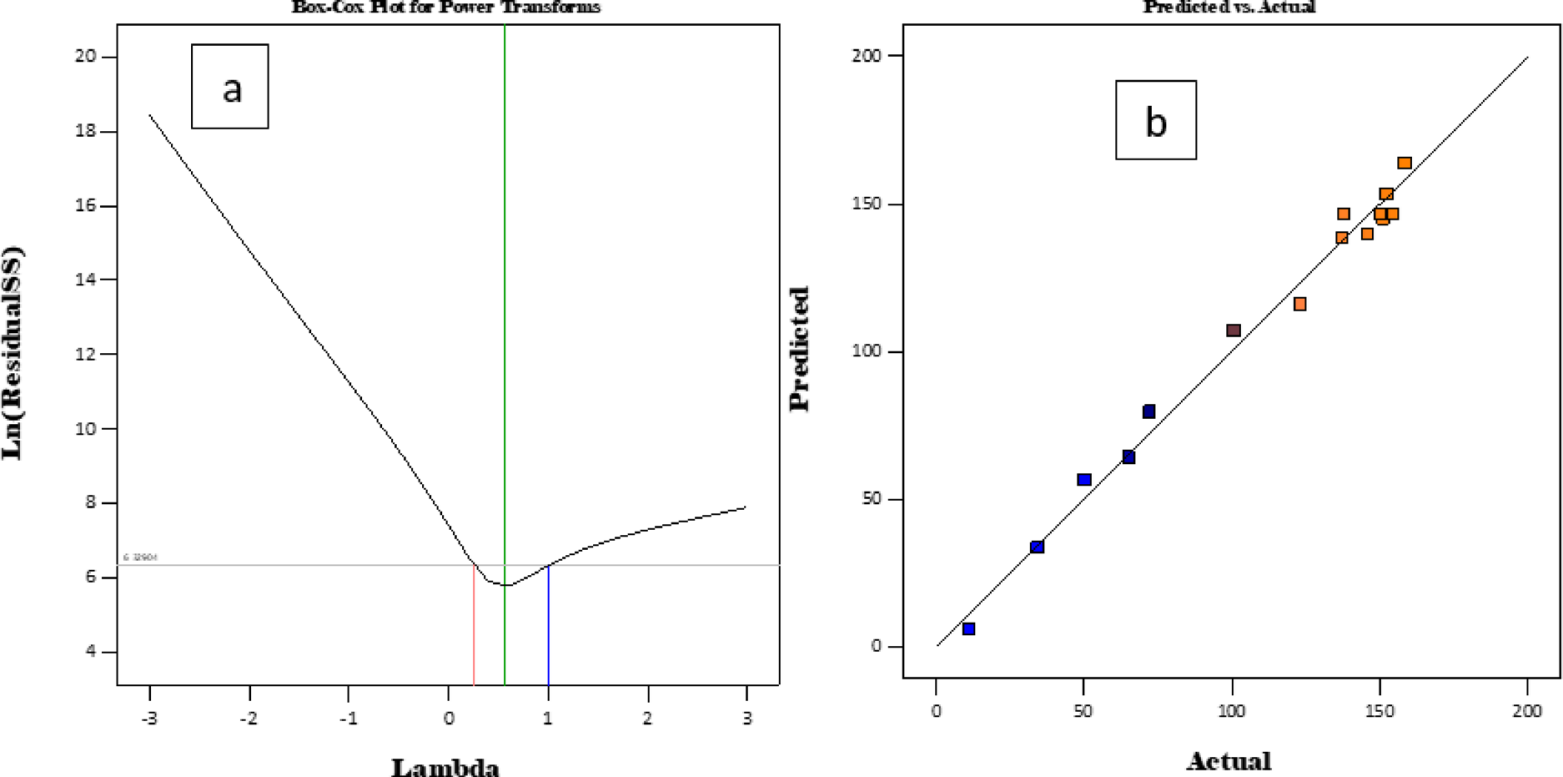
Plots of the Box-Cox and the predicted versus the actual data.

This was determined by the box-cox plot and the actual versus predicted values as shown in Fig. 14.

Figure 14a demonstrates that the Box–Cox analysis yields a λ close to 1, indicating that transformation was not necessary when the adsorption capacity is being determined. The data dispersion shown in Fig. 14b gives a correlation that is linear, signifying that the output is closely aligned with the anticipated response values.

### Residuals and cook’s number

**Fig. 15:**
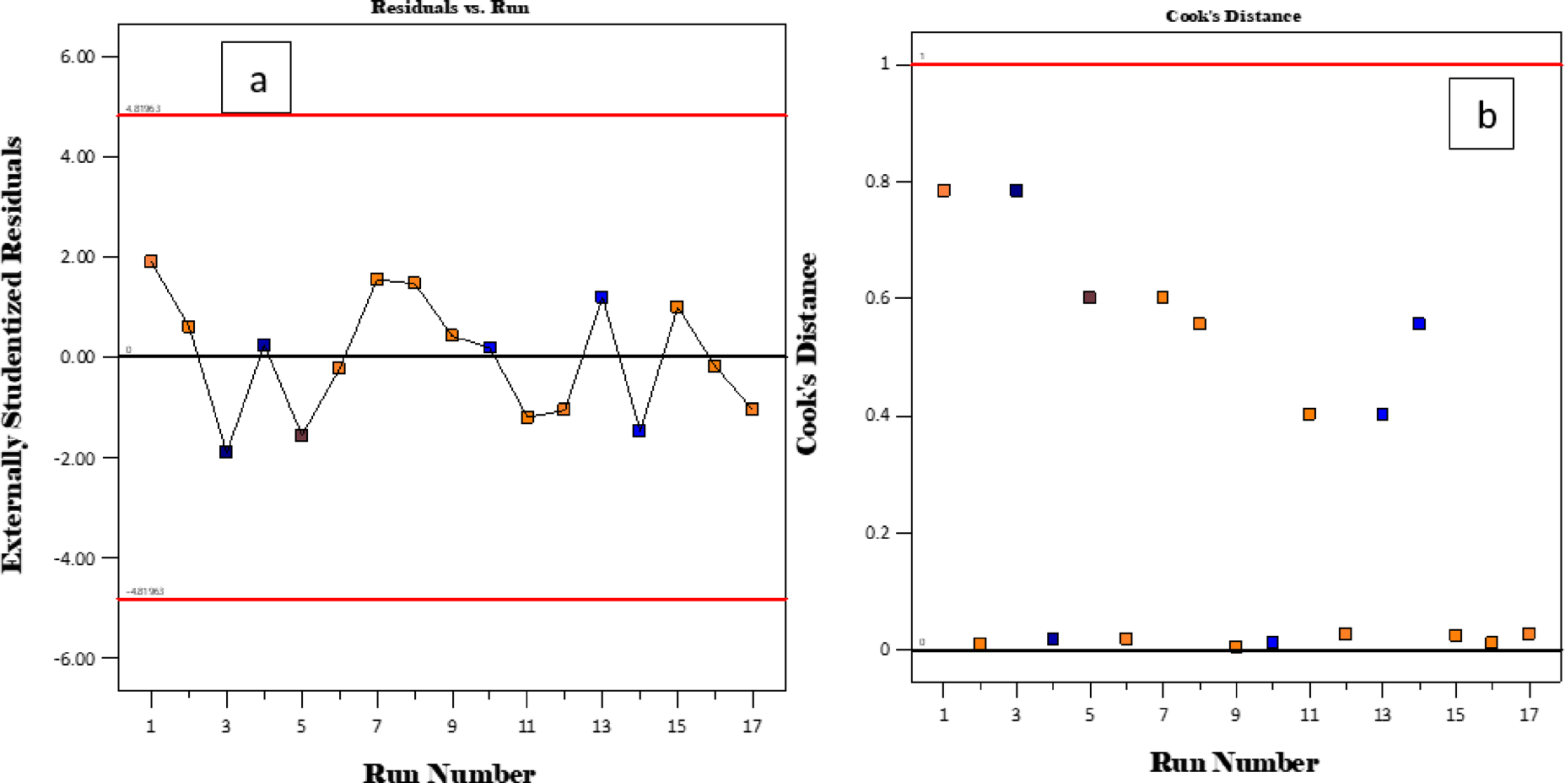
Plots of run number against predicted values, the residuals, and Cook’s distance.

Figure 15 shows a plot of residuals and cook’s distance against the run number.

Additionally, the graphs of residuals versus run number and Cook’s distance anticipated values for adsorption capacity are presented in Fig. 15 (a and b). The cook’s distance shows that the majority of data points were below 0.2, demonstrating the adequacy of the model to model the adsorption capacity (Fig. 15b).

**Fig. 16:**
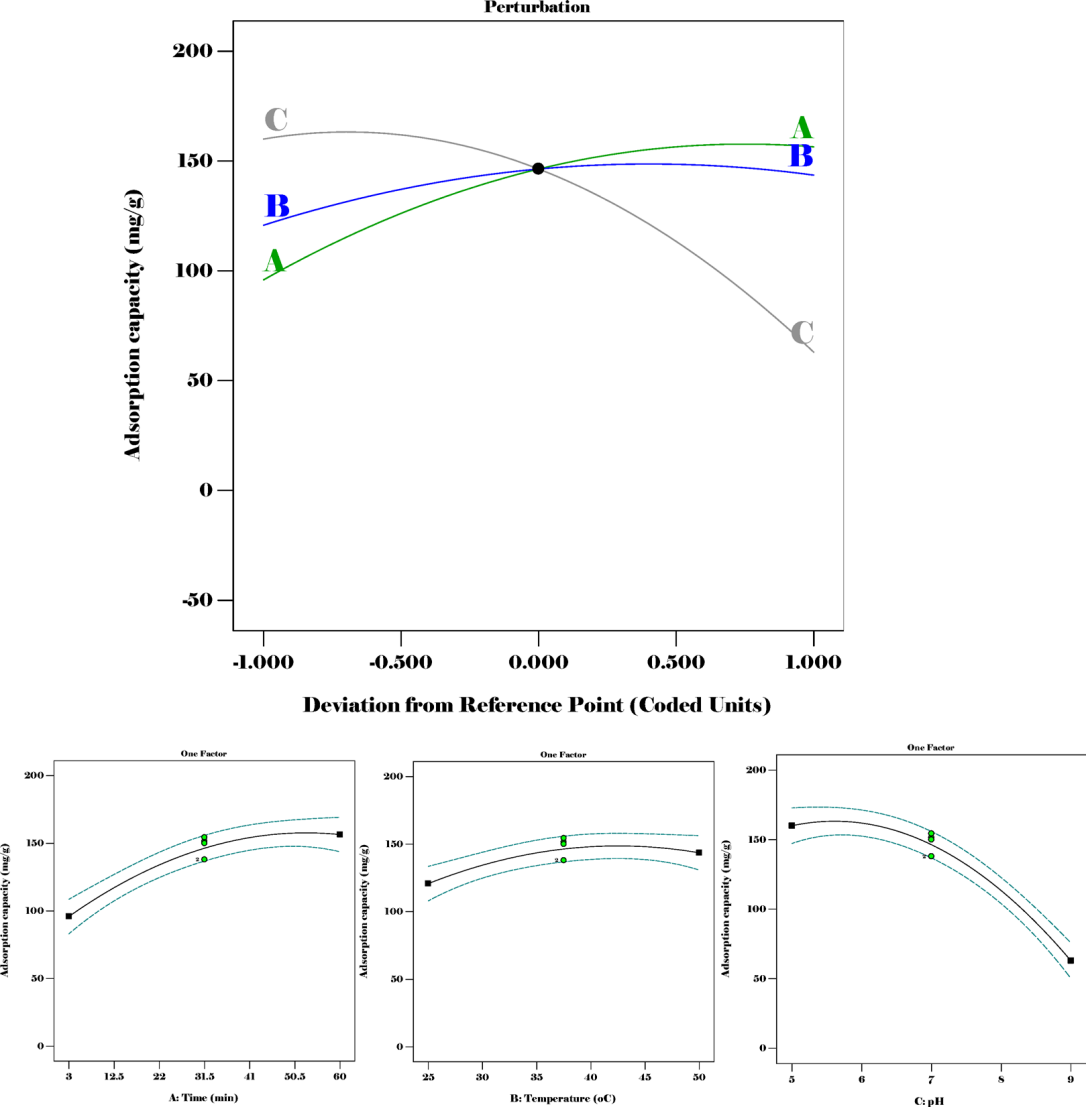
A plot of perturbation and the individual interaction of the input against the output.

The fluctuation of the three parameters concerning adsorption was further depicted by a graph illustrating the perturbation and interaction among the individual components and the adsorption capacity, as demonstrated in Fig. 16.

From this, the three input variables were important in understanding the adsorption capacity. The increase in temperature caused a significant increase in the adsorption capacity; correspondingly, the pH had a similar effect.

### Desirability function

The desirability function introduced by Derringer and Suich in 1980 serves as a method for optimising multiple responses at once. The process involves transforming each experimental result into a desirability score (di) that spans from 0 to 1. A score of 1 indicates that the desired outcome for that response was successfully met, whereas a score of 0 signifies that the response did not fall within the acceptable parameters.

The formula for calculating desirability is presented in Eq. 3.

3 $$D=\left({d}_{1}\times{d}_{2}\times\dots\times{d}_{n}\right)\frac{1}{n}=\left({\prod}_{i=1}^{n}{d}_{1}\right)\frac{1}{n}$$

**Fig. 17:**
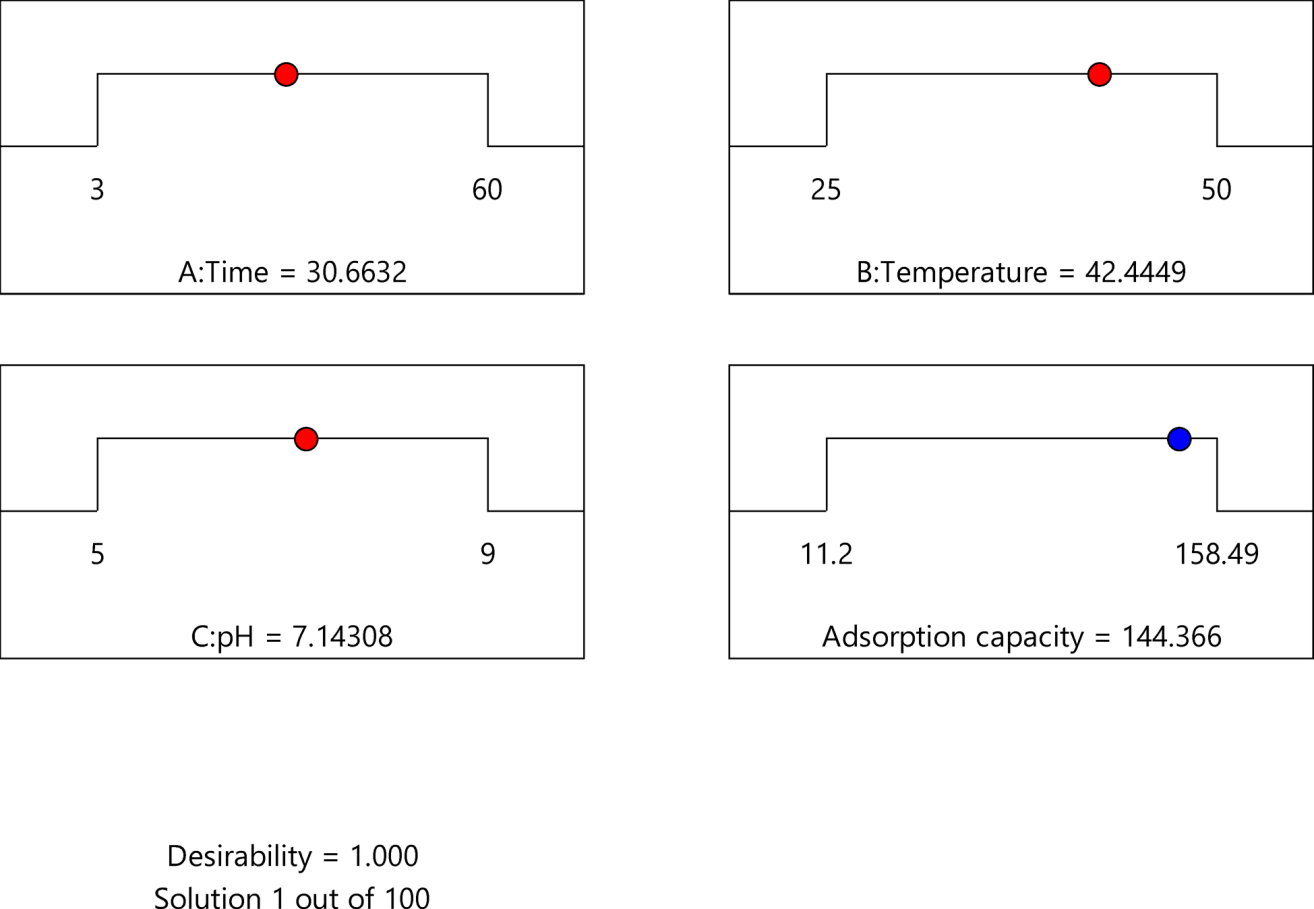
Desirability function for the best process variables.

D represents total desirability, n denotes the response number, and d_i_ signifies individual desirability, respectively. This investigation sought to determine the ideal circumstances for enhancing desirability. A desirability function was utilised to concurrently optimise the temperature, pH, and time. This method enabled us to identify the optimal combination of these factors for attaining the best adsorption capacity. The analysis indicates that optimal removal was attained with a time of 30.663 min, a pH of 7.143, and a temperature of 42.45 °C, as shown in Fig. 17.

Figure 17 demonstrates that the optimal adsorption capacity of 144.366 was attained at a total desirability of 1.000. These data are obtained under precise conditions: pH of 7.14, temperature of 42.45 °C, and duration of 30.67 min. The desirability function of 1.000 indicated optimal conditions for maximum adsorption capacity. The desirability of 1.000 confirms the model’s acceptance and applicability, signifying that it is an efficient method for establishing optimal conditions. The results closely matched the projected outcomes, confirming the model’s adequacy and validity. The outcomes of the optimization for adsorption are displayed in Table 6. The findings demonstrate that the maximum adsorption capacity is attained at a pH of 7.14, a duration of 30.67 min, and a temperature of 42.45 °C, resulting in an optimal adsorption capacity of 144.366 mg/g.

**Table 6:** RSM optimization data for optimum conditions.

Number	Time	Temperature	pH	Adsorption capacity	Desirability	Selected
1	30.663	42.445	7.143	144.366	1.000	*
2	31.500	50.000	9.000	64.054	1.000	
3	31.500	25.000	9.000	33.416	1.000	
4	6.800	39.167	8.733	35.162	1.000	
5	59.500	44.121	5.002	156.714	1.000	
6	31.500	37.500	7.000	146.378	1.000	
7	3.000	25.000	7.000	56.305	1.000	
8	3.000	50.000	7.000	107.047	1.000	
9	60.000	50.000	7.000	139.695	1.000	
10	31.500	50.000	5.000	153.304	1.000	
11	31.500	25.000	5.000	138.356	1.000	
12	60.000	25.000	7.000	144.852	1.000	
13	3.000	37.500	5.000	115.949	1.000	
14	27.773	28.610	8.620	60.333	1.000	
15	34.996	44.125	7.792	127.378	1.000	
16	7.149	43.416	5.540	133.100	1.000	
17	33.473	48.184	6.533	155.464	1.000	

**Fig. 18:**
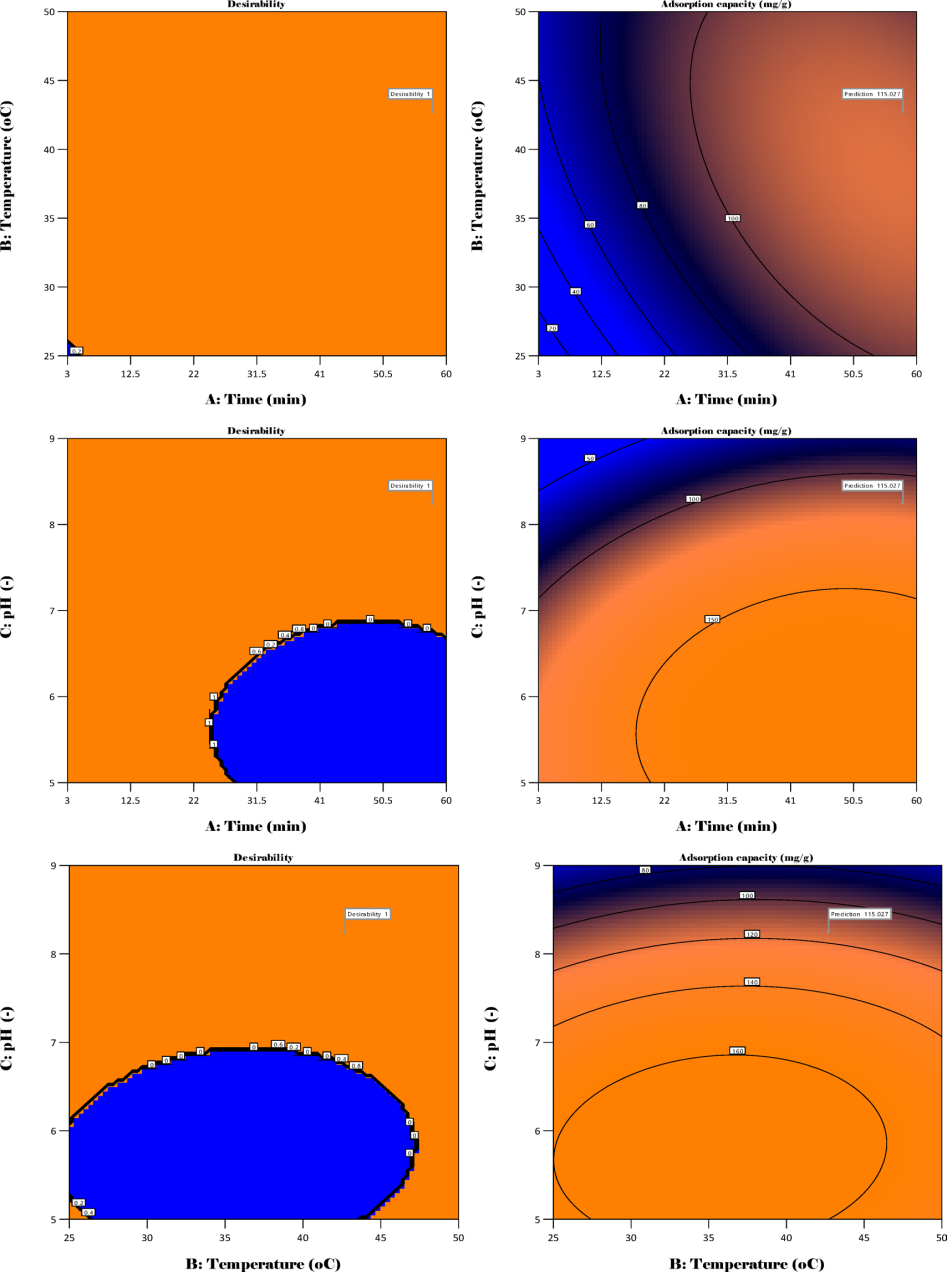
3D and 2D surface and contour plots for the desirability at optimum conditions.

Numerical optimization was attained by predicting the best conditions for several parameters, including pH, temperature, and time, for adsorption utilizing the RSM model. The model’s primary purpose was to attain maximal adsorption. Figure 18 depicts the surface response plot in conjunction with the contour plot of desirability under ideal conditions. The results demonstrate that the correlation among all three input variables significantly impacts the adsorption capacity.

### Optimization using ANNs

**Fig. 19:**
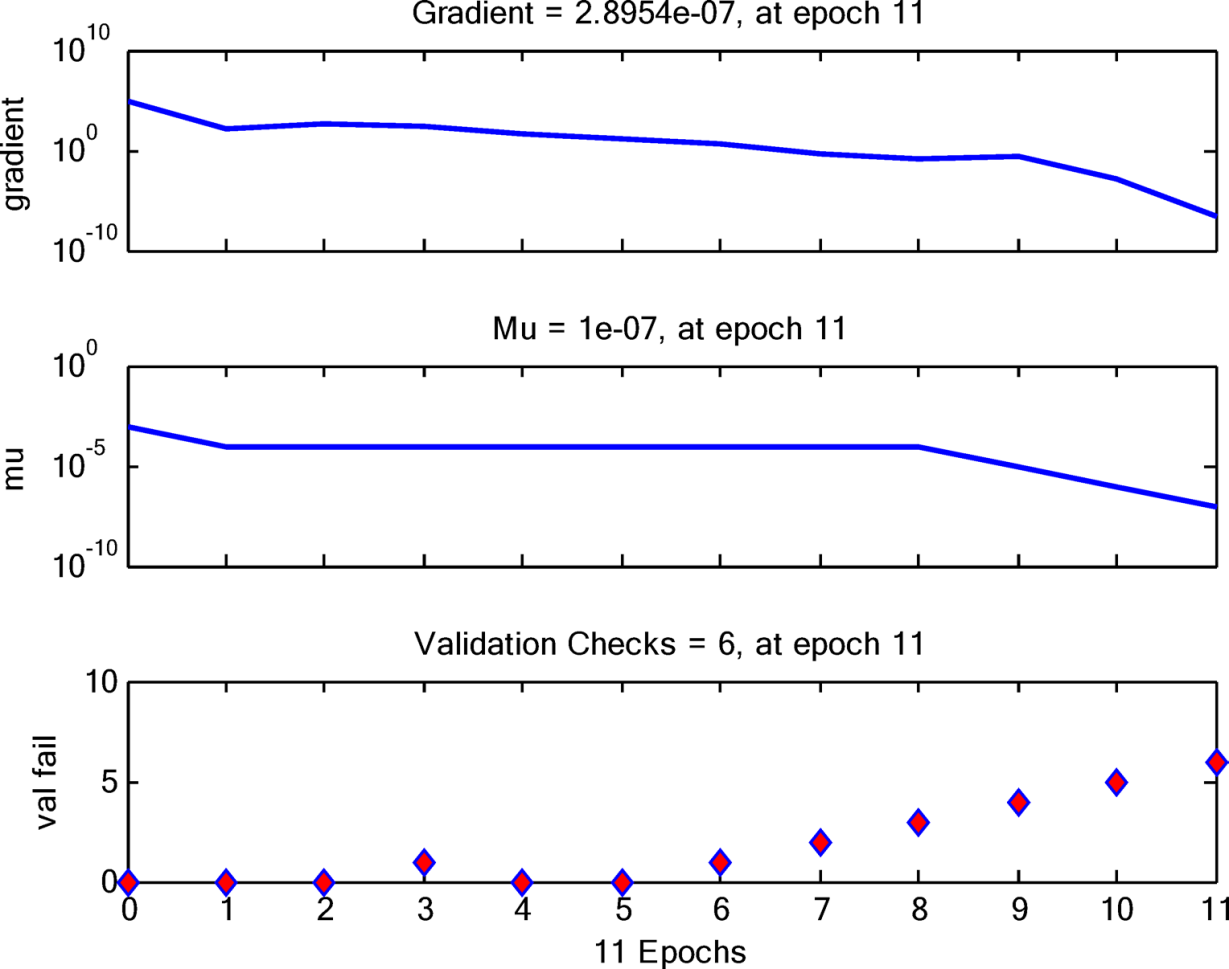
A plot of the training sets against the failure.

To optimize the model, the input neurons and output neurons are maintained constant, while the hidden layer neurons are modified^73^. The model’s efficacy was evaluated by analyzing the variation in the neurons^74^. The MSE of the validation test exhibited a notable variability over the range of selected neurons utilized in the network training set, as illustrated in Fig. 19.

The plot of the training sets against failure is produced utilizing the gradient, mu, and validation, which are based on the epochs. The gradient exhibits a progressive decline across the Epochs, whilst the mu remains rather constant with a little decrease following the 8th Epoch. Finally, the validation plot indicates a failure at the 11th Epoch, as seen by the increase following the ideal point.

**Fig. 20:**
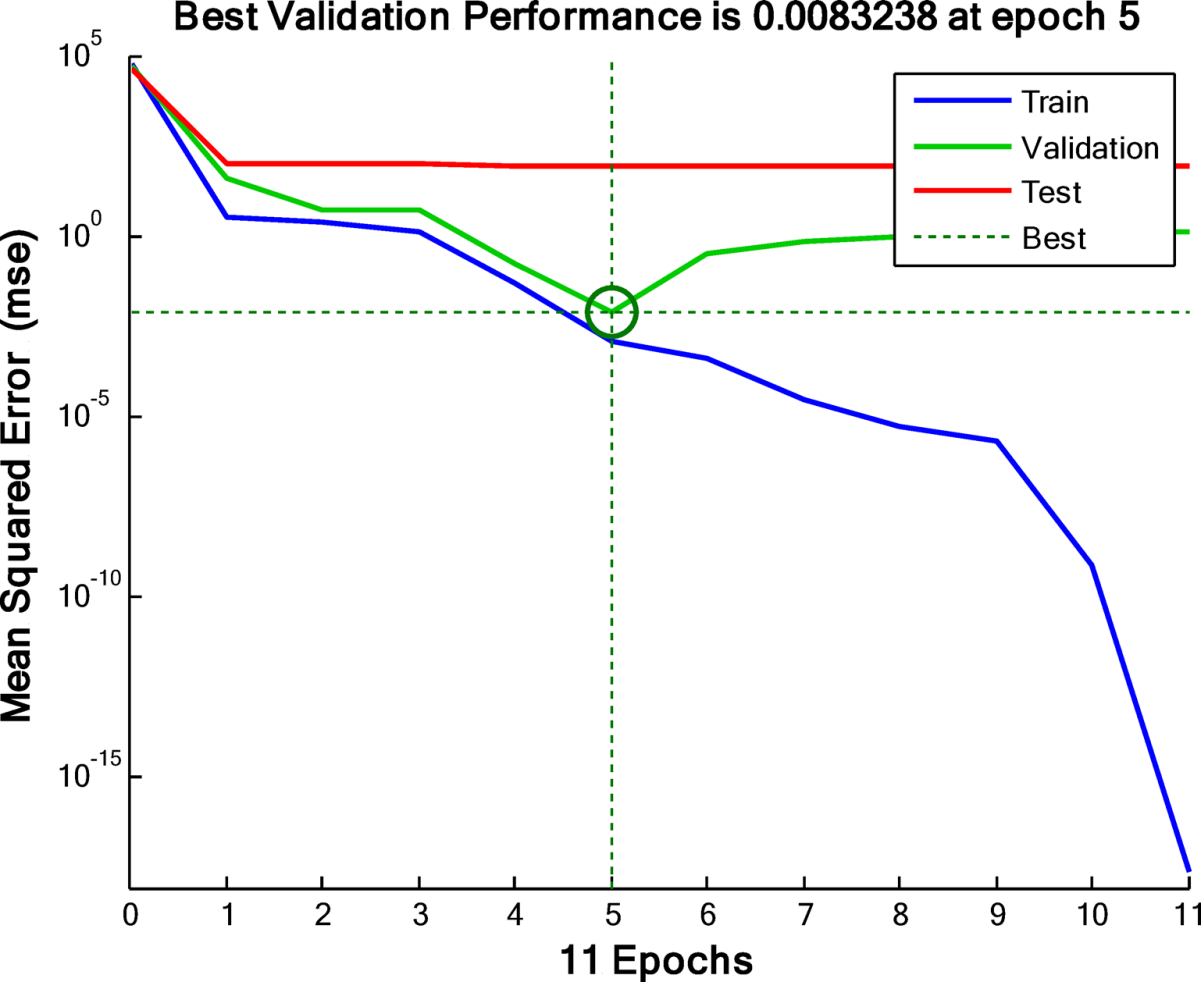
A plot of the best validation performance.

Figure 20 illustrates a graph of the mean squared error in relation to the epoch utilized for validating, training, and testing the model data to optimize network performance.

The performance of the training set is evaluated using a plot of mean squared error (MSE) against the number of Epochs for the system at optimum conditions, which gives information regarding the validation of the model^53^. The plot shows that the best performance validation at the appropriate MSE is about 0.0083238 at the 5th Epoch iteration. A small MSE suggested that the training network experienced no overfitting problems. It is worth noting that an increase in the test curve before the first rise in validation would imply overfitting.

**Fig. 21:**
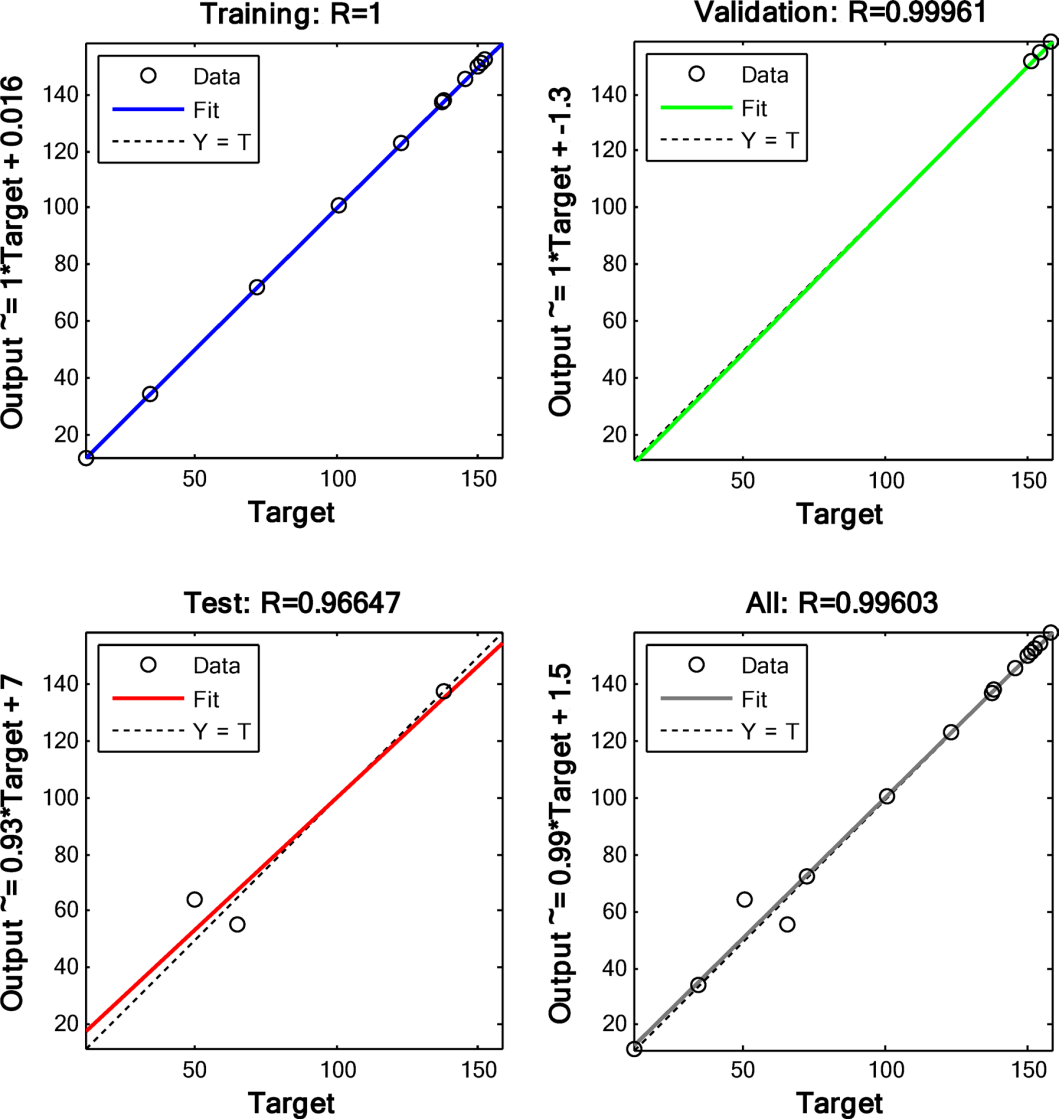
Regression graphs of validation, training, overall, and test.

Figure 21 shows the regression plot of the validation, training, overall, and testing of the process variables.

Figure 21 shows the plot of the validation, training, test, and overall regression of the normalized data sets. The initial step involved conducting data normalization. The data obtained was as follows: training (1), testing (0.99961), validation (0.96647), and total (0.99603). It is observed that all four data sets had values close to 1, and thus were able to effectively model and predict the adsorption capacity.

### ANFIS modeling

**Fig. 22:**
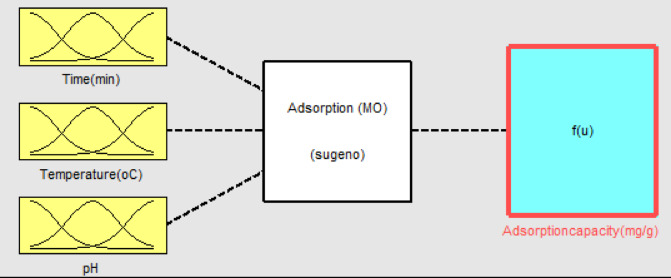
A diagram of the Sugeno fuzzy inference system.

The experimental and predicted data were further verified using the ANFIS model. It was designed using both a gradient and a least squares method that was based on a hybrid learning approach. The system efficiency of the fuzzy model was then improved through the normalization of data^53^. Figure 22 depicts the development of the Sugeno-type Fuzzy Inference System utilizing the grid partition approach.

**Fig. 23:**
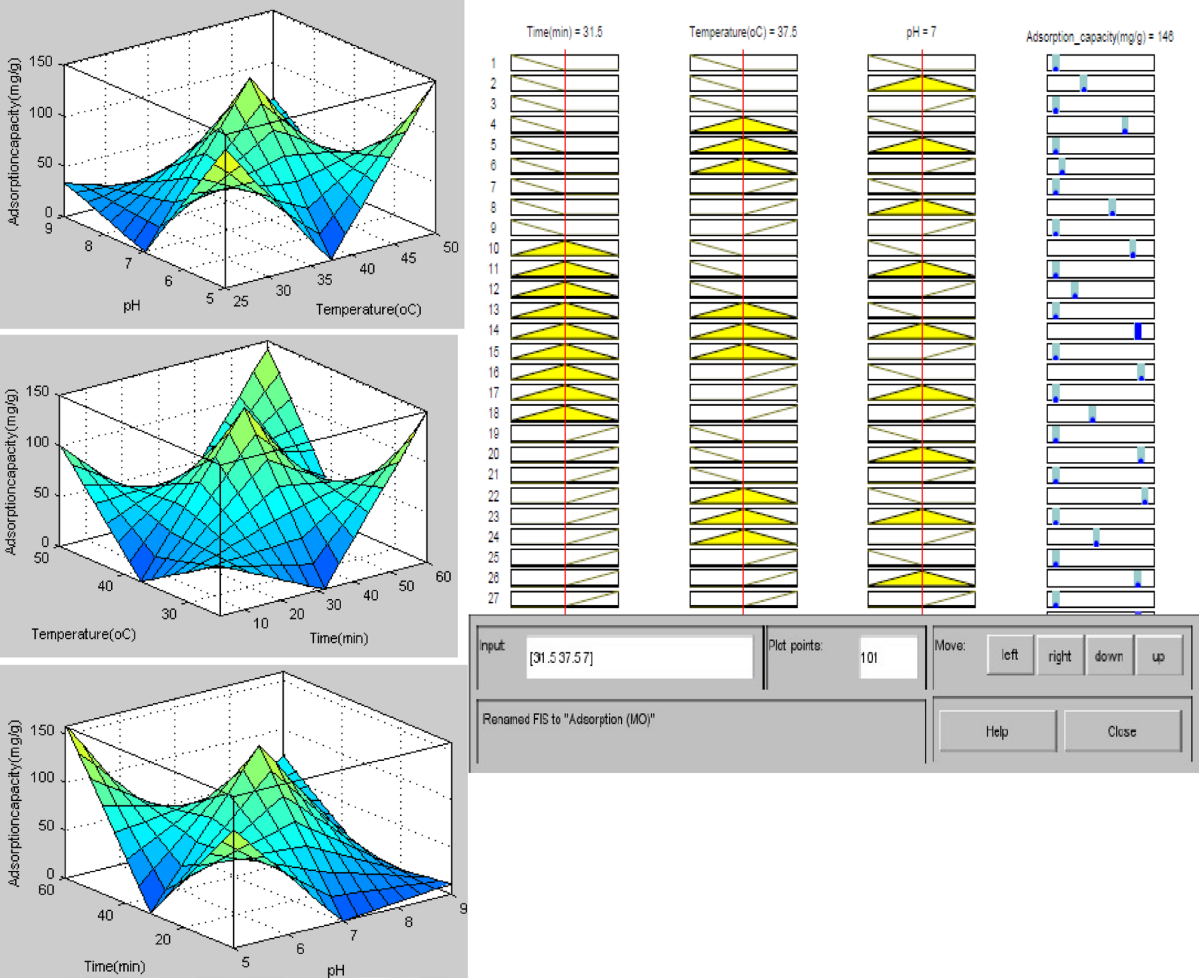
Surface and rule view of the interaction between the input and output variables.

Additionally, to comprehend the interaction among input factors, a surface plot was created, as depicted in Fig. 23.

Figure 23 shows the three-dimensional plots and a rule view that demonstrate the interaction between pH (7), time (31.5 min), and temperature (37.5 °C). This suggests a notable interaction between pH and temperature, resulting from the increased surface area, improved diffusion of MB into the internal pores of AC, and strong interactions with functional groups^75^. The relationship between temperature and time indicates a significant correlation between these two variables, as evidenced in both the 3D and rule views. An observation revealed that a temperature of 37.5 °C achieved at 31.5 min resulted in the best adsorption capacity. This outcome arises from prolonged contact duration coupled with a significant surface area, but also an increase in the kinetic energy, which results in more interaction between the adsorbate and adsorbent. The effect of pH on time was analyzed using a 3D plot and the rule view. The optimization of pH and time on the overall adsorption capacity played a significant role. The adsorption capacity is improved with extended contact time, attributed to the establishment of robust bonds between the adsorbent and the adsorbate. At neutral pH, the interaction among competing ions is negated, leading to a much better adsorption capacity. The pH of the media, therefore, had a very big effect on the adsorption capacity. The model identifies pH as the primary determinant variable in this study, significantly impacting both time and temperature, which in turn affects the adsorption capacity.

### RSM, ANN and ANFIS comparative analysis

The models are evaluated to precisely identify the one with the highest predictive capability as presented in Table 7.

**Table 7:** Optimization and prediction power of the three machine learning models.

Run	Time (mins)	Temperature (°C)	pH	Adsorption capacity (mg/g)	RSM	ANN	ANFIS
1	3	37.5	5	123.2	116.0	123.2	123.2
2	31.5	37.5	7	151.5	146.4	151.4	146.4
3	60	37.5	9	72.2	79.5	72.2	72.2
4	31.5	50	9	65.2	64.1	55.3	65.2
5	3	50	7	100.7	107.1	100.7	100.7
6	31.5	25	5	137.2	138.4	137.1	137.2
7	60	25	7	151.2	144.9	151.1	151.2
8	60	50	7	145.8	139.7	145.8	145.8
9	31.5	37.5	7	150.0	146.4	150.0	146.4
10	31.5	25	9	34.3	33.4	34.3	34.3
11	60	37.5	5	158.5	163.7	158.5	158.5
12	31.5	37.5	7	138.0	146.4	137.9	146.4
13	3	37.5	9	11.2	6.0	11.2	11.2
14	3	25	7	50.2	56.3	64.3	50.2
15	31.5	37.5	7	154.4	146.4	154.5	146.4
16	31.5	50	5	152.4	153.3	152.4	152.4
17	31.5	37.5	7	137.9	146.4	137.9	146.4

The comparative analysis in the table indicates that all three models demonstrate strong predictive capabilities, with values closely aligning with the actual results and showing only minimal differences. It was noted that ANFIS demonstrated the highest predictive accuracy, while RSM followed, and ANN exhibited the lowest performance^53^.

**Fig. 24:**
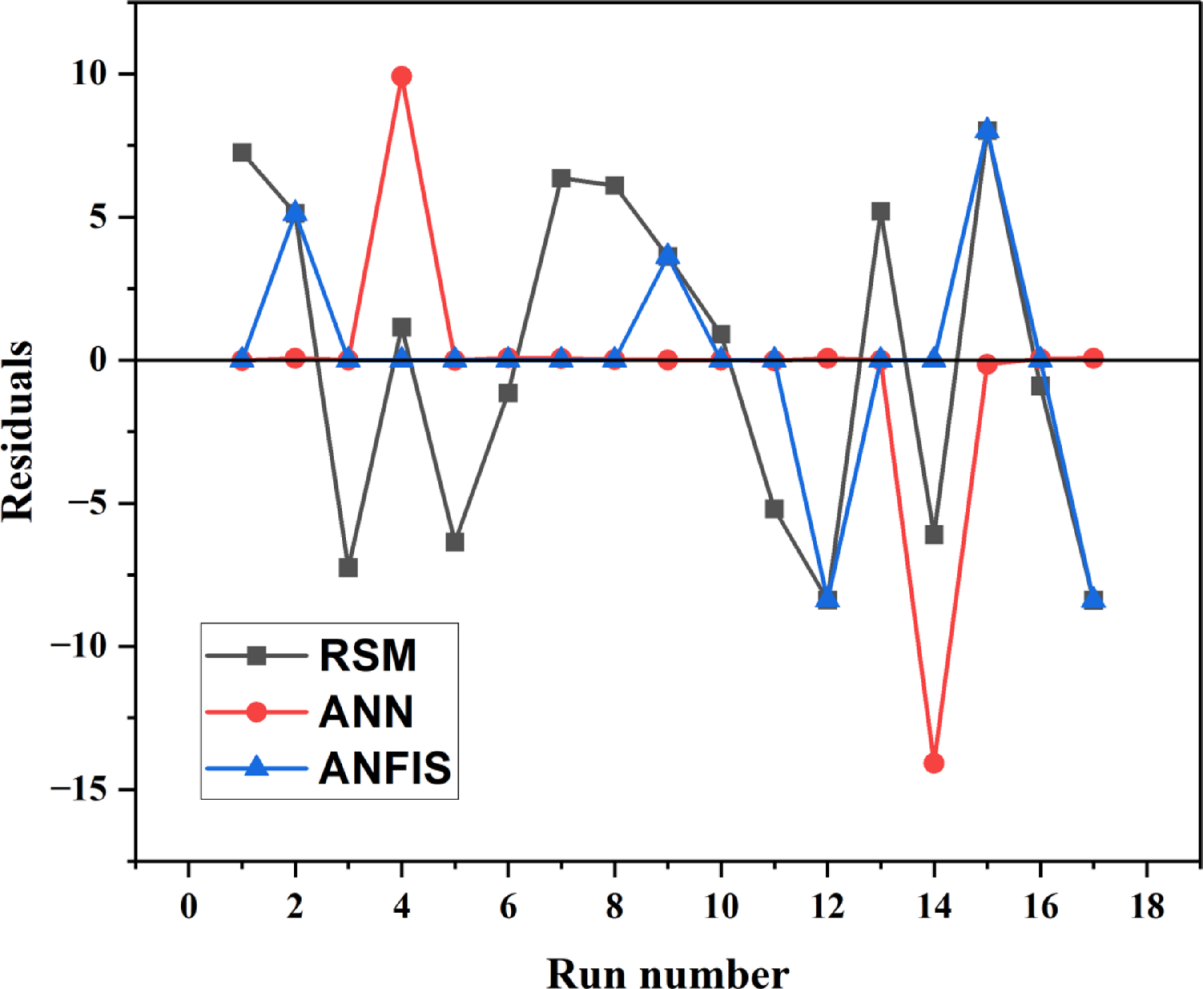
Comparison of the residuals against the run number of BBD, ANN, and ANFIS.

This study also conducted a residual plot analysis to evaluate the differences among the three models, as shown in Fig. 24.

Figure 24 shows that the residual error associated with the ANFIS-predicted data approaches zero^84^. Additionally, the plot indicates that only a limited number of data points from the ANFIS are significantly distant from the central line of the residual plot. It is crucial to observe that the residual values of RSM and ANN exhibited a comparable trend, with only minor variations from those of ANFIS^76^. The residual plots indicate that the ANFIS model produced predictions closely aligned with the actual data, thereby reinforcing its superiority over both RSM and ANN.

**Fig. 25:**
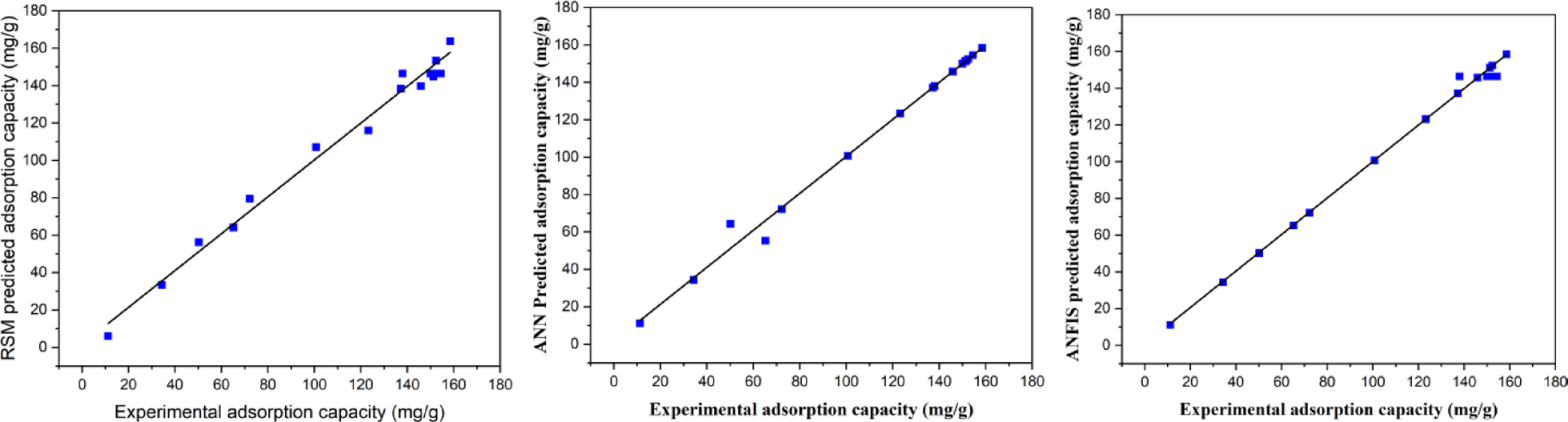
A graph of regression showing plots for experimental against predicted data.

Lastly, this study examined the accuracy of the three models and their capability to predict and enhance the process through the use of performance metrics. Figure 25 shows a plot of regression that depicts the distribution of the data sets of the experimental and predicted data sets.

The performance metrics utilized for model evaluation comprised MAE, R^2^, Pearson’s correlation coefficient, MSE, Adjusted R^2^, and RMSE. The metrics adhere to the principle that a lower value indicates superior predictive and optimization capabilities. The six performance indices for the predicted values of the models are presented in Table 8.

**Table 8:** Performance measures for RSM, ANN, and ANFIS.

Entry	Performance Indices	ANFIS	RSM	ANN
1	R-Square	0.9934	0.9921	0.9849
2	Adj. R-Square	0.9930	0.9916	0.9839
3	Pearson’s r	0.9967	0.9960	0.9924
4	MSE	16.1773	19.3908	36.9531
5	RMSE	4.0221	4.4035	6.0789
6	MAE	0.0000	0.2353	0.0006

The performance metrics of the six functions indicate that ANFIS was the top-performing model, then RSM, and ANN last. The choice of the model is based on the data obtained from the analysis of the evaluation indices. To show very high predictive power, models with lower error function values are best suited for the data since this shows very low deviations from the actual data. The mean square error determines the model’s accuracy based on the lower values obtained which indicate smaller prediction errors. It showed that the ANFIS and RSM had very high accuracy compared to ANN. The root mean square error was well in agreement with the three models; however, ANFIS had a better edge compared to ANN and RSM. A study on the mean average error was also carried out, and it had low values for the three models, which further shows the predictive accuracy. The R^2^ and the adjusted R^2^ were also determined to investigate the correlation, which also showed that ANFIS was superior, followed by RSM and then ANN. ANFIS had, high R^2^ (0.9934), Adjusted R^2^ (0.9930), Pearson’s r (0.9967), and low MSE (16.1773), RMSE (4.0221), and MAE (0.000). ANN with R^2^ (0.9921), Adjusted R^2^ (0.9916), Pearson’s r (0.9960), MSE (19.3908), RMSE (4.4035), and MAE (0.2353) was second. BBD with R^2^ (0.9849), Adjusted R^2^ (0.9839), Pearson’s r (0.9924), MSE (36.9531), RMSE (6.0789), and MAE (0.0006). This provides additional evidence that the ANFIS model demonstrates greater reliability in predicting and optimizing the adsorption capacity.

### Kinetic studies

**Fig. 26:**
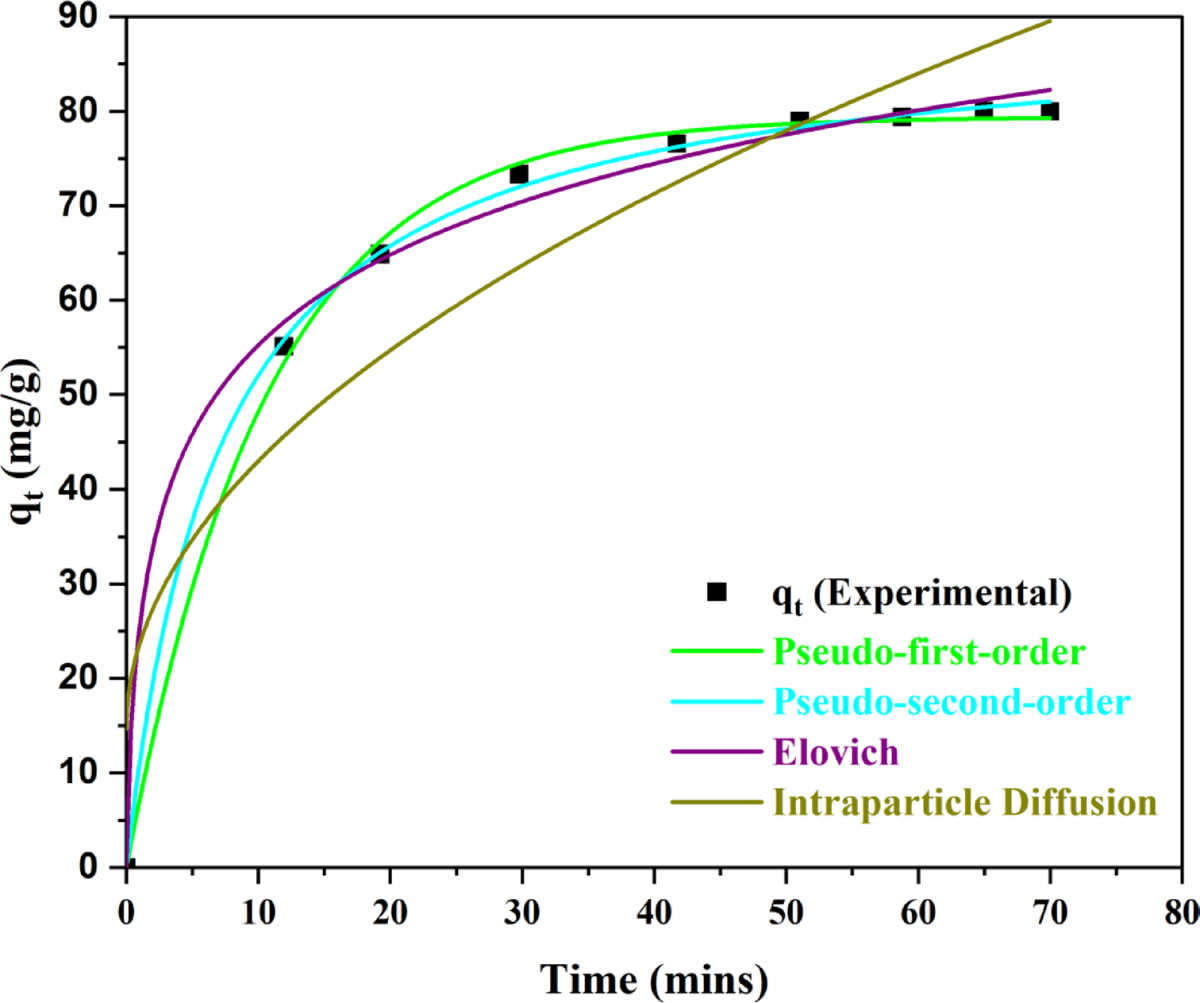
Kinetic models for MO adsorption.

The time studies for the analysis of the interaction of MO and BP-AC were assessed at different time intervals and illustrated in Fig. 26.

Table 9 shows the kinetic models and their constants used to determine the model that best describes the adsorption mechanism.

**Table 9:** Kinetic models used in MO removal based on their parameters.

Entry	Model	Parameters	*R* ^2^
1	PSO	q_e_ = 89.3134 (mg/g) k_1_ = 0.00156 (min^− 1^) χ^2^ = 0.5921	0.9992
2	PFO	q_e_ = 79.3914 (mg/g) k_2_ = 0.09338 (g/mg.min) χ^2^ = 1.2865	0.9983
3	Elovich	a_E_ = 70.9092 mg/(g min) b_E_ = 0.07141 mg/(g min) χ^2^ = 3.7463	0.9951
4	Intraparticle Diffusion (IDF)	K_diff_ = 6.3469 (mg/g.min^0.5^) C = 33.1784 (mg/g) χ^2^ = 11.9991	0.9274

Table 9 shows the constants for PFO, Elovich, PSO, and intraparticle diffusion which are used to determine the rate limiting step and mechanism of adsorption demonstrated high correlation coefficient values. The IDF model with an R^2^ value of 0.9274, K_diff_ of 6.3469 (mg/g.min^0.5^) and C of 33.1784 (mg/g) described the rapid diffusion characterized with low mass resistance on the surface. The data obtained from the Elovich (R^2^ = 0.9951) shows that the process of adsorption occurred on surfaces that were energetically stable. Lastly the data obtained from PFO (R^2^ = 0.9983) and PSO (R^2^ = 0.9992), was used to elucidate the adsorption of MO to occur by both chemisorption and physisorption respectively. The chi-square was used to determine the model with the best quality which was important in making the best choice^77^. The PSO had the lowest chi square value, and as such, chemisorption was the dominant mechanism of adsorption.

### Isotherm studies

**Fig. 27:**
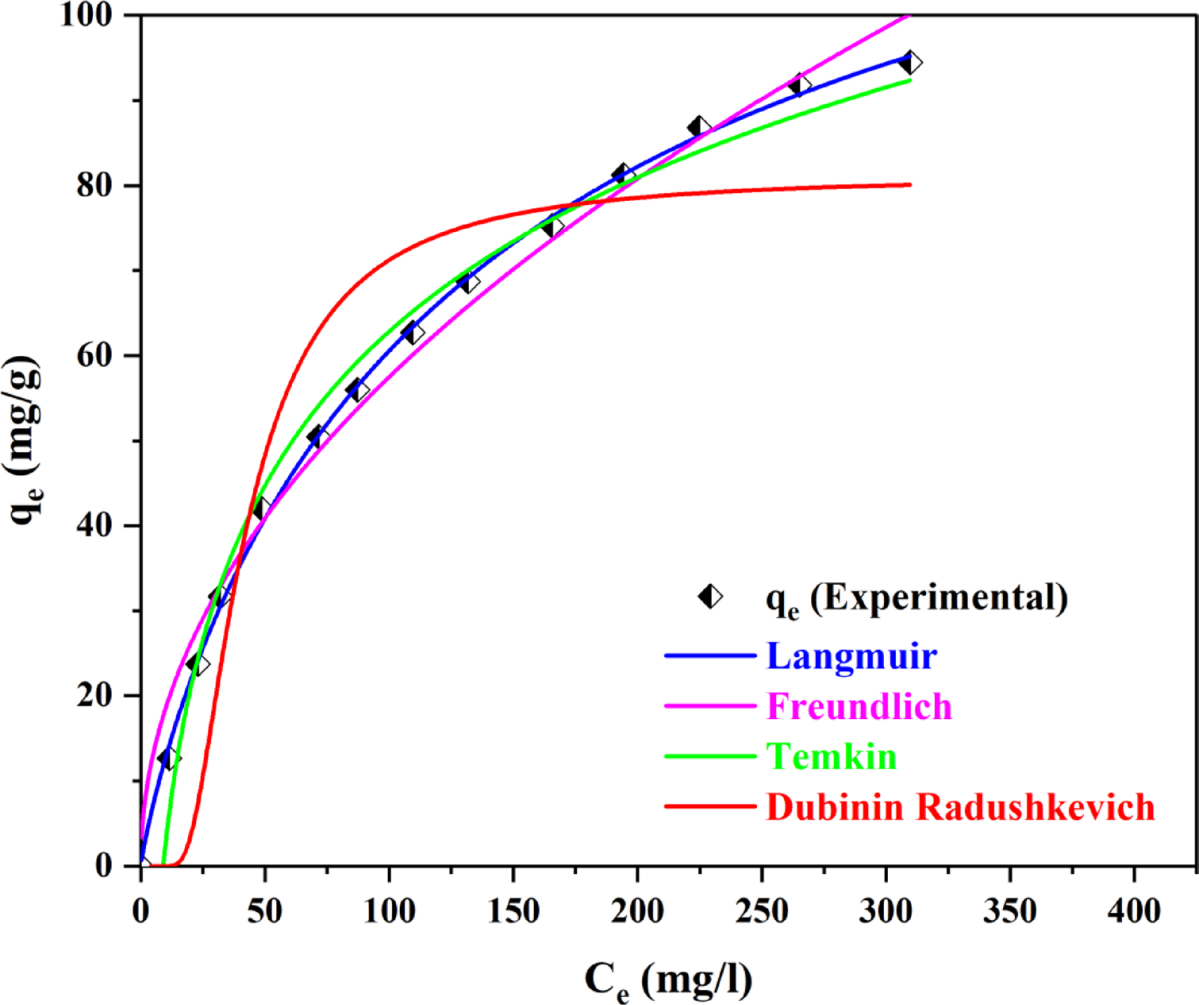
Isotherm models for MO equilibrium adsorption.

To comprehend the equilibrium interaction, isotherm models were examined as illustrated in the Fig. 27.

The graphs of the isotherm models exhibit a strong relationship with the data obtained from the experiments. The detailed analysis of the isotherm models is presented in Table 10.

**Table 10:** Isotherm models used in MO equilibrium studies.

Entry	Isotherm	Parameters	*R* ^2^
1	Freundlich	k_f_ = 56.9857 (mg/g) (L/mg)^1/n^, 1/*n* = 0.4912 χ^2^ = 12.4437	0.9857
2	Temkin	A = 0.1107 (L/g) B = 26.1317 (mg/l) χ^2^ = 9.5159	0.9877
3	Langmuir	k_L_ = 0.01301 (L/mg) q_max_ = 144.3319 (mg/g) χ^2^ = 1.2430	0.9985
4	Dubinin-Radushkevich	q_m_ = 81.1865 (mg/g) K_DR_ = 0.2919 (mol/kJ)^2^ E = 0.5336 (kJ/mol) χ^2^ = 22.6249	0.8348

Table 10 illustrates that the four isotherm models have good correlation coefficients which are used to understand the equilibrium interaction. The 1/n ratio of 0.4912, is a strong indicator that MO and BP-AC have strong forces of attraction for each other^78^. This shows that multilayer surfaces were responsible for the adsorption process, which characterizes the significance of variability. The q_max_ of 144.3319 mg/g in the Langmuir model shows the capacity of the BP-AC to adsorb MO and thus describes the presence of monolayer adsorption. Temkin with an R^2^ value of 0.9877 and its corresponding constants shows a reduction in the heat of adsorption when MO gets attached onto BP- AC. Ultimately, the D-R model exhibited an R² value of 0.8348, which generally signifies the distribution of Gaussian energies on surfaces characterized by heterogeneity^79^. The low chi-square value indicates that the Freundlich model is the best fit, suggesting that adsorption occurs on multilayer surfaces.

### Adsorption mechanism

**Fig. 28:**
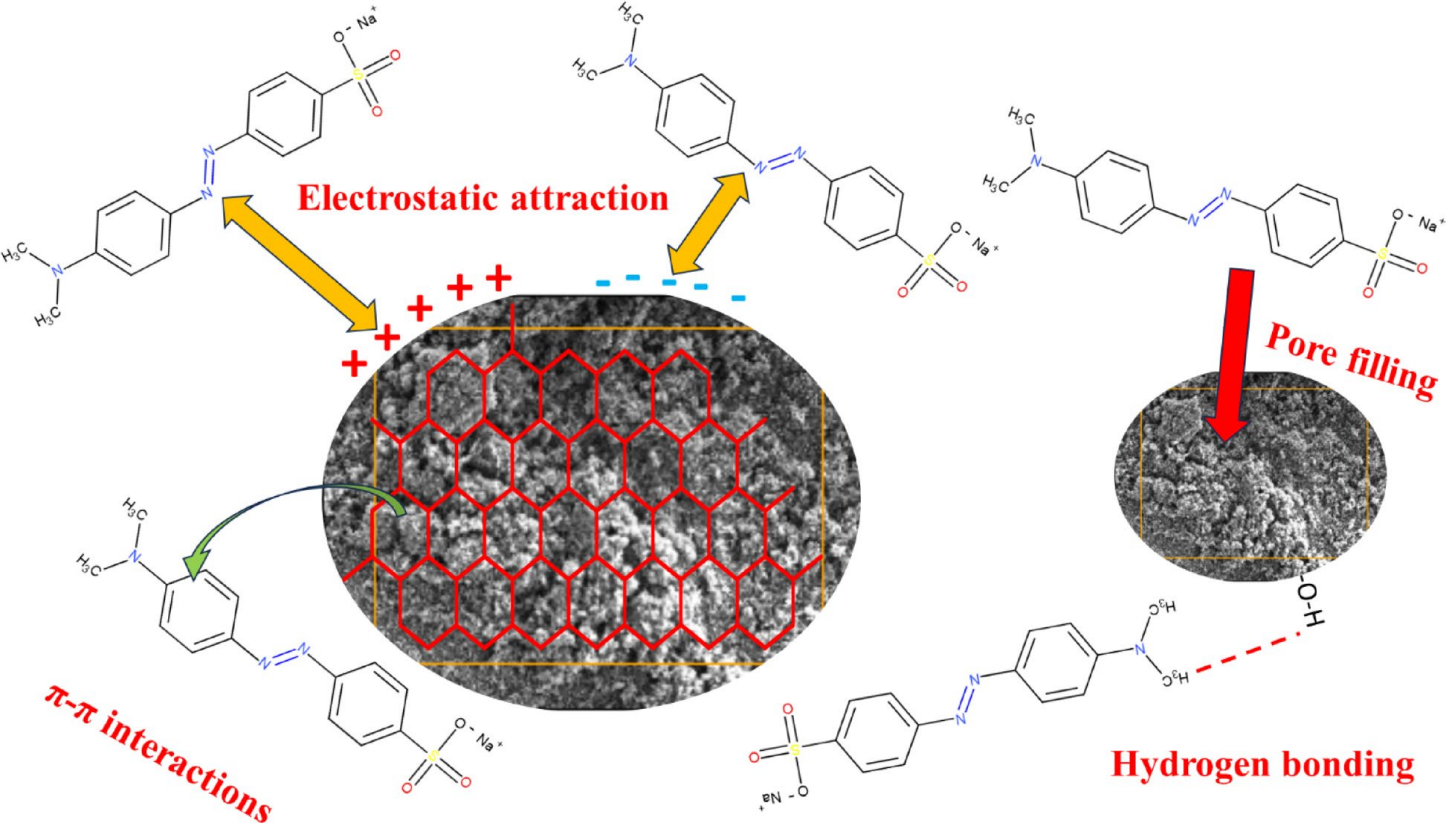
**.** An overview of the adsorption mechanisms for the removal of MO.

The adsorption mechanism, which involves the bonding of methyl orange and BP-AC, was elucidated using characterization techniques such as BET, SEM, PXRD, and FTIR. From the analysis of the characterization, it was determined that the possible mechanisms might be due to hydrogen bonding, electrostatic attraction, pore filling, and π-π interactions, as shown in Fig. 28.

Figure 28 shows that the mechanism of adsorption of MO on plastic waste activated carbon was due to pore filling, electrostatic attraction, hydrogen bonding, and π-π interactions. Initially, the N_2_ physisorption isotherm shows the presence of a large surface area with adequate pore sizes onto which MO gets adsorbed, accounting for pore filling, electrostatic attraction, and π-π interactions. SEM showed the porous nature of the adsorbent, which describes the adsorption of MO in the pores of BP-AC with some π-π interactions due to the presence of a large area onto which the MO can interact. Using XRD, the mechanism of adsorption was due to hydrogen bonding, due to the presence of an amorphous structure, which creates the availability of hydroxyl groups, which gives rise to the type of bonding. Lastly, FTIR showed the presence of carbonyl, aromatic, and hydroxyl functional groups, which can aid π-π interactions, electrostatic attraction, and hydrogen bonding. The mechanisms were also verified by the adsorption isotherms and kinetics, where observations were made regarding the presence of chemisorption on energetically stable heterogeneous surfaces.

### Application in real water samples

**Fig. 29:**
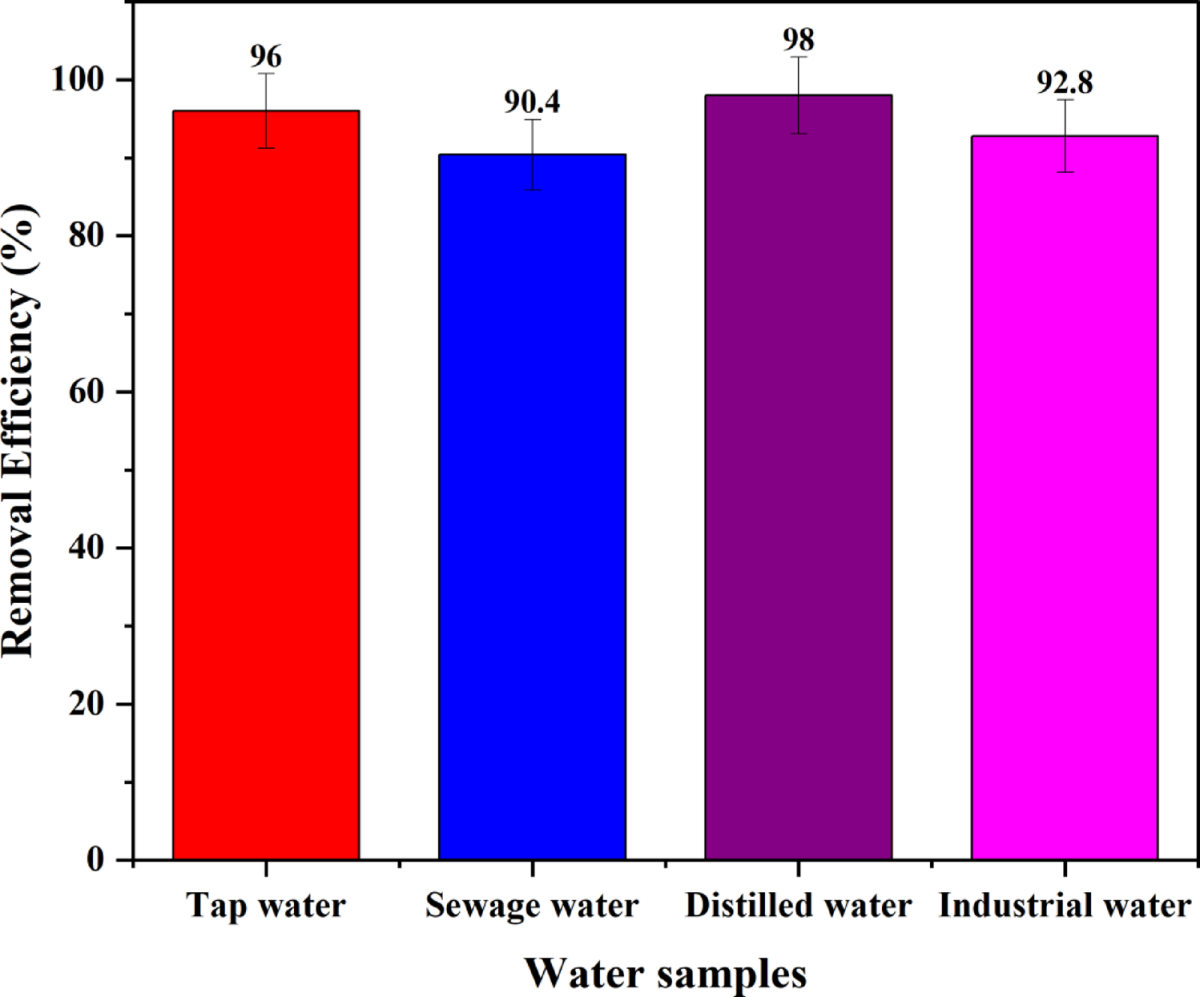
Applicability of the adsorbent in real water samples.

The applicability of BP-AC on the adsorption of methyl orange was studied using four different water samples, which were sewage, tap, industrial, and distilled water. The four water samples were spiked with 100 mg/l of methyl orange to investigate the applicability of the adsorbent in real-world processes. Before the adsorption studies were done, the water was passed through a 0.45 μm sieve. The percentage removal of methyl orange by BP-AC was seen to be above 90.3, as shown in Fig. 29.

Figure 29 shows that samples containing industrial and sewage water have low efficiency (90.4%) and (92.8%), respectively, due to the presence of other ions, which compete for surface functional groups of BP-AC.

### Reusability

**Fig. 30:**
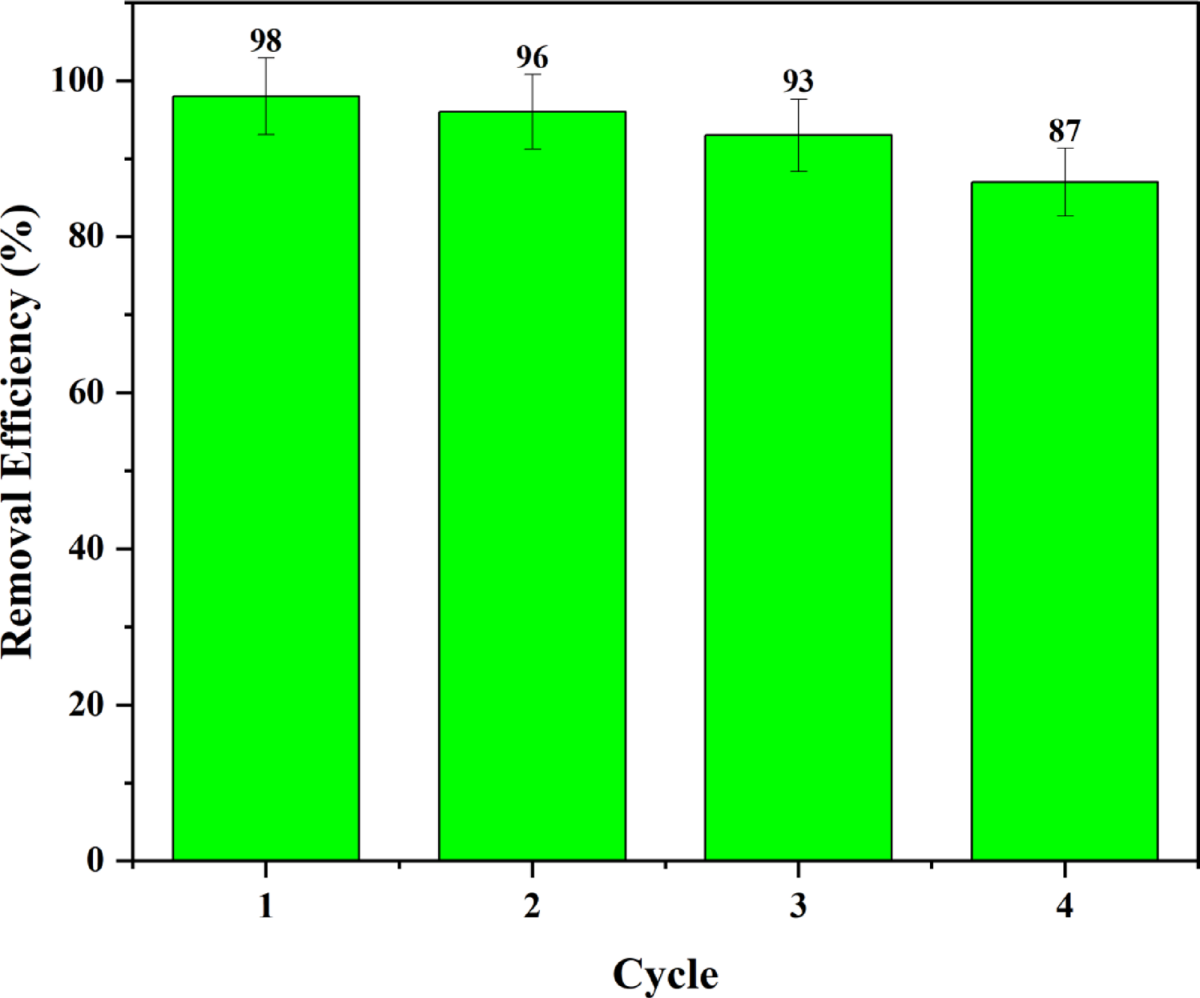
Reusability cycles of BP-AC.

The study examined the reusability of BP-AC for the removal of methyl orange, aiming to elucidate its economic viability, practical applicability, and mechanistic framework. Four cycles were obtained for the reuse of the adsorbent at optimal conditions. The adsorbent was reused by applying NaOH (0.1 M) solution to assess its desorption capability. The adsorbent’s reusability and stability diminished progressively after four cycles, as illustrated in Fig. 30.

Figure 30 shows the percentage removal of methyl orange after the first four cycles. The observed reduction in removal efficiency might be caused by strong adsorbate/adsorbent interaction in which the active sites are occupied by methyl orange molecules. It is observed that the desorption is low even after NaOH is added to the adsorbent.

### Comparison of BP-AC with previous studies

Various writers have documented studies on the elimination of methyl orange to assess removal efficiency. The comparative analysis focused on the adsorption capacity q_max_ (mg/g) of the adsorbent. Table 11 illustrates the comparability between this study and prior research.

**Table 11:** Comparability analysis of this study and previous literature on MO removal.

Entry	Adsorbents	q_max_ (mg/g)	Reference
1	Crosslinked chitosan-glyoxal/TiO_2_ nanocomposite (CCG/TNC)	416.1	^80^
2	Modified orange peel (MOP)	321.2	^81^
3	Boron nitride nanosheets (BNNSs)	575.0	^82^
4	Orange peel-titanium dioxide nanoparticles (OPe-TiO₂)	111.1	^83^
5	MgAl layered double hydroxide	539.1	^84^
6	MnO_2_- Modified Diatomite	1.66	^85^
7	BP-AC	144.3	This study

Table 11 shows that the synthesized banana peel-activated carbon has the ability to perform better than some previous studies from literature. The observed adsorption capacity of 144.3 mg/g is achieved within 24 min. From the analysis of previous studies on the removal of MO from water, it can be concluded that plastic waste-activated carbon has a high edge in the adsorption process.

## Conclusion

This study successfully demonstrated the synthesis of activated carbon from invasive banana peels, highlighting its potential as a sustainable and effective adsorbent for water treatment. Characterization of the activated carbon was conducted using scanning electron microscopy, Fourier transform infrared spectroscopy, thermogravimetric analysis, powder X-ray diffraction, and BET. Adsorption capacity studies were carried out using methyl orange, whose input variables were predicted and optimized using Box-Behnken design (BBD), artificial neural networks (ANN), and adaptive neural fuzzy inference system (ANFIS). Using the BBD, the optimal conditions were identified at a pH of 7.14, a temperature of 42.45 °C, and a time of 30.67 min, achieving an impressive predicted adsorption capacity of 144.4 mg/g. ANN (137.9 mg/g) was also optimized using the process variables from the BBD, and the optimal conditions that occurred were assessed through the study of the best validation performance, the test of curve plots, and the mean squared error of the validation. In addition, an ANFIS (146.0 mg/g) was utilized, and it gave optimal conditions at a pH of 7, time of 31.5 min, and a temperature of 37.5 °C. Performance metrics were used to determine the model with the best predictive capacity. The ANFIS model had superior optimization and predictive accuracy compared to ANN and BBD. The pseudo-second-order and the Freundlich models were used to model the process and, as such, showed that MO adsorption occurred on heterogeneous multilayer surfaces of BP-AC. This comparative analysis confirms ANFIS as the most reliable and accurate model for predicting and optimizing the adsorption capacity of banana peel-derived activated carbon for methyl orange removal, offering a promising avenue for sustainable water purification.

## Data Availability

The datasets used and/or analyzed during the current study are available from the corresponding author on reasonable request.

## Code availability

The codes used in this current study are deposited in a zenodo public repository: 10.5281/zenodo.18823019.
